# A novel monoclonal antibody reveals the enrichment of NADPH oxidase 5 in human splenic endothelial cells

**DOI:** 10.1038/s41598-023-44018-5

**Published:** 2023-10-11

**Authors:** Zsolt Szeles, Gábor L. Petheő, Bence Szikora, Imre Kacskovics, Miklós Geiszt

**Affiliations:** 1 Department of Physiology, Faculty of Medicine, Semmelweis University, PO Box 259, 1444 Budapest, Hungary; 2 ImmunoGenes Ltd., Budakeszi, Hungary

**Keywords:** Biochemistry, Biological techniques, Cell biology, Molecular biology, Physiology

## Abstract

Members of the NOX/DUOX family of NADPH oxidases are responsible for regulated ROS production in diverse cells and tissues. Detection of NOX/DUOX proteins at the protein level remains an important challenge in the field. Here we report the development and characterization of a novel anti-NOX5 monoclonal antibody, which recognizes the human NOX5 protein in both Western blot, immunocytochemistry, and histochemistry applications. With the help of the antibody we could successfully detect both heterologously and endogenously expressed NOX5 in mammalian cells. Furthermore, we could also detect NOX5 protein in the human spleen, testis, and ovary. Immunohistochemical studies on human testis revealed that NOX5 localized to spermatogenic cells. This expression pattern was also supported by the result of in silico analysis of single-cell RNA sequencing data that indicated that NOX5 protein is present in developing spermatids and spermatocytes. Mature spermatozoa, however, did not contain detectable NOX5. In the human ovary, both immunostaining and single-cell RNA sequencing suggest that NOX5 is expressed in interstitial fibroblasts and theca cells. We also analyzed vascular cells for the presence of NOX5 and we found that NOX5 expression is a fairly specific feature of splenic endothelial cells.

## Introduction

Reactive oxygen species (ROS) have important physiological roles in living organisms, and disturbed ROS metabolism contributes to the development of various pathological states^1^. There are multiple cellular sources of ROS, but the members of the NOX/DUOX family of NADPH oxidases are now thought to be the main sources of regulated ROS production^2–4^. There are seven NOX/DUOX enzymes in humans and they are expressed in a wide variety of tissues and cells. Genetic evidence now supports their function in host defense, vasoregulation, hormone biosynthesis, otoconia formation, and development^5^. One of the least characterized members of the family is NADPH oxidase 5 (NOX5). Similar to other NOX/DUOX enzymes, NOX5 is also a heme-containing membrane protein with six transmembrane helices^6^. NOX5 contains calcium-binding EF-hands, which are located in the N-terminal region of the protein. This structure highly resembles the domain composition of plant NOXes^7^, and based on this similarity NOX5 is often described as the most “ancient” among the family members. The presence of EF-hands provides the structural basis of NOX5 activation, which is induced by a rise in cytosolic calcium. Human NOX5 was originally identified in the testis and spleen and it was subsequently detected in other cells and tissues including the esophagus and blood vessels^8^. Unlike other members of the NOX/DUOX family, NOX5 is absent from the genome of rodents. The explanation for this unusual evolutionary feature is currently unknown, but it essentially excludes the possibility to explore the physiological role of NOX5 in rodent models. On the other hand, NOX5 is present in rabbits^9^, and its expression pattern resembles that of the human homolog. Our lab has recently reported the development of NOX5-deficient rabbits and the identification of their higher sensitivity to cholesterol diet-induced atherosclerosis^10^.

A major obstacle in NOX/DUOX research is the lack of antibodies that can specifically detect the different isoforms in cells and tissues where they are naturally expressed. The availability of specific and sensitive antibodies would be essential to explore the tissue distribution and subcellular localization of different NOX/DUOX isoforms. An excellent illustration of this problem was recently provided by Diebold et al., who characterized commercially available and custom-made anti-NOX/DUOX antibodies^11^. It is a cause for concern that several of the tested antibodies failed the rigorous testing procedure which should essentially exclude them from future studies.

In this article, we report the development and characterization of a mouse, monoclonal, anti-NOX5 antibody that specifically recognizes both heterologously and endogenously expressed human NOX5. With the help of this antibody, we report for the first time the detection of the human NOX5 protein in the human testis and spleen. We also show that the NOX5 protein is expressed in the human ovary. Despite the high sensitivity of the antibody, we found no evidence for the expression of the NOX5 protein in spermatozoa, thus contradicting previous observations of NOX5 expression in gametocytes. Furthermore, we found no evidence for NOX5 expression in vascular endothelial and smooth muscle cells, a finding which is supported by data from qPCR experiments and single-cell RNA sequencing.

## Results

### Development of a novel, monoclonal anti-NOX5 antibody

Detection of NOX/DUOX enzymes at the protein level is crucial for getting a better understanding of their localization and functions. After several unsuccessful trials to detect NOX5 with commercially available antibodies, we decided to develop a monoclonal antibody against human NOX5. The N-terminal fragment of human NOX5, containing 167 amino acids was produced in *E. coli* in the form of a GST fusion protein. We used this antigen to immunize hemizygous transgenic mice which carry five copies of the cDNA encoding the α-chain of bovine FCGRT gene^12^. These transgenic animals were previously described to produce antibodies with higher sensitivity/specificity when compared to wild-type mice^12^. For selecting hybridoma clones, which produce anti-NOX5 antibodies, we used a His-tagged version of the NOX5 antigen, thereby we could filter out the clones which produced antibodies against the GST portion of the antigen. We identified multiple positive clones, and the one (IMG-1E10) which produced the antibody with the highest sensitivity was used for ascites production and antibody purification.

### Establishment and characterization of a NOX5-expressing cell line

**Figure 1:**
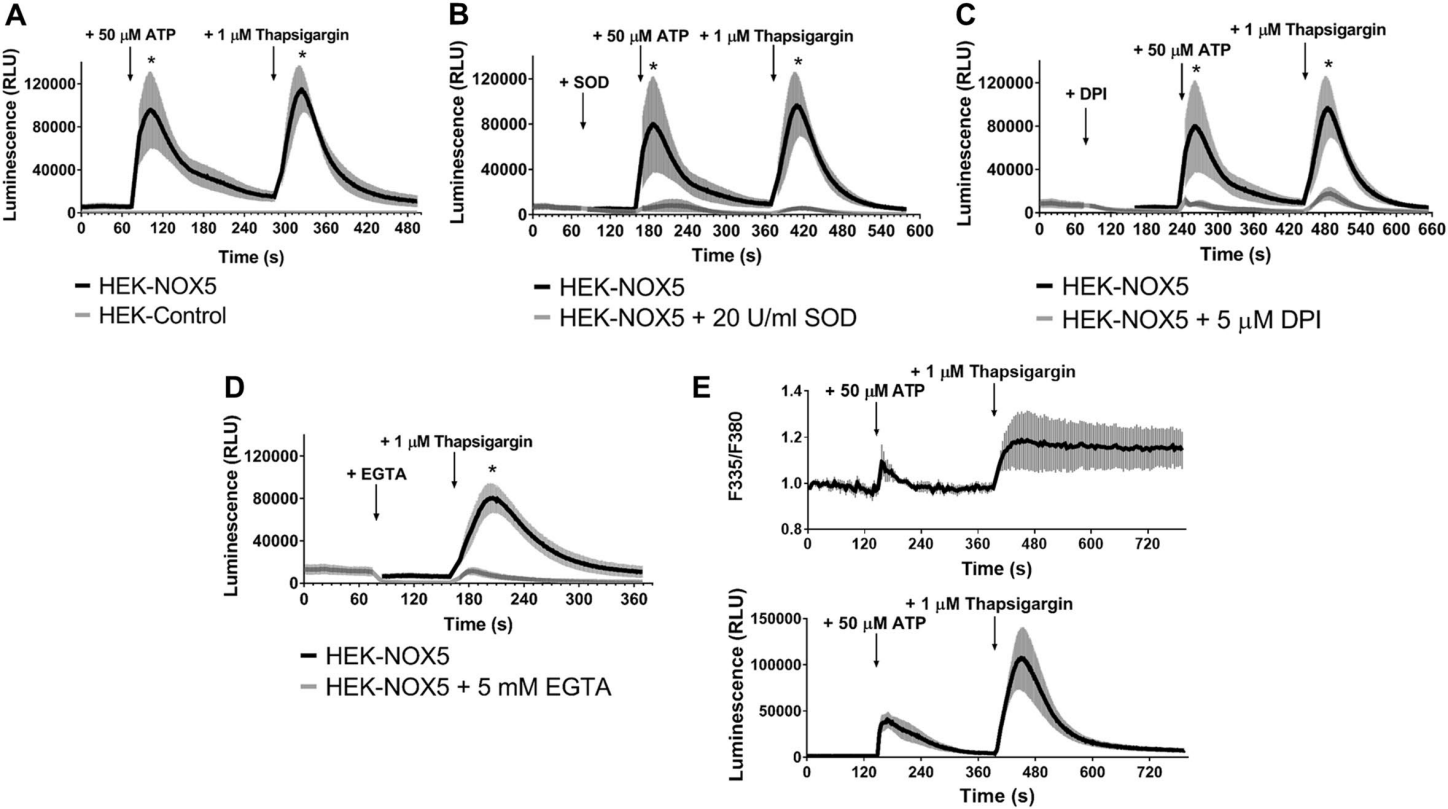
Functional characterization of NOX5 overexpressing HEK293T cells. (**A**–**D**) Detection of extracellular superoxide signal, produced by NOX5 overexpressing HEK293T cells. (**A**) Stimulation of superoxide production by receptor agonist ATP and SERCA inhibitor thapsigargin. (**B**) Reduction of superoxide signal by addition of superoxide dismutase (SOD). (**C**) Inhibition of NOX5 enzyme by diphenyleneiodonium chloride (DPI). (**D**) Reduction of superoxide production by adding EGTA to chelate the extracellular Ca^2+^. (**E**) Temporal relationships between the Ca^2+^ and superoxide signals in NOX5 overexpressing HEK293T cells after different stimuli. The upper part of the figure shows the Ca^2+^ level changes in the cells, while the lower part shows the extracellular superoxide level. *n* = 3–4, mean ± SEM.

To create a test system for the characterization of the antibody, we stably expressed NOX5 in HEK293T cells. To confirm that the transfected cells contain a functional enzyme, we ran a series of tests to characterize the ROS production by NOX5-expressing cells. Since NOX5 is activated by an increase in intracellular calcium concentration^6^, we used the purinergic receptor agonist ATP and the SERCA-inhibitor thapsigargin (TG) as stimuli, respectively. As depicted in Fig. 1A, ATP (50 µM) stimulation resulted in an increase in superoxide production in NOX5-expressing cells, as determined by the Diogenes chemiluminescence reagent. After a decline in the chemiluminescence, the SERCA inhibitor thapsigargin (1 µM) also induced a superoxide response. Importantly, neither compound elicited ROS response in control cells, transfected with empty vector (HEK-Control), (Fig. 1A). The chemiluminescence signal was efficiently quenched by the addition of superoxide dismutase, thus proving that superoxide production was responsible for the observed signal (Fig. 1B). Superoxide production was also efficiently inhibited by 5 µM diphenyleneiodonium (DPI), a non-specific, but potent inhibitor of NADPH oxidases (Fig. 1C). In the case of thapsigargin, we also determined the role of extracellular calcium in the evoked ROS response. Figure 1D demonstrates, that chelating of extracellular calcium by EGTA inhibited the TG-stimulated superoxide production. It is interesting to note, that both ATP and thapsigargin elicited transient ROS responses, although the kinetics of calcium signals evoked by them are remarkably different (Fig. 1E). While ATP induced a transient calcium signal, the SERCA-inhibitor thapsigargin evoked a stable increase in intracellular calcium concentration (Fig. 1E).

### Detection of heterologously expressed NOX5 by a novel, anti-NOX5, monoclonal antibody

**Figure 2:**
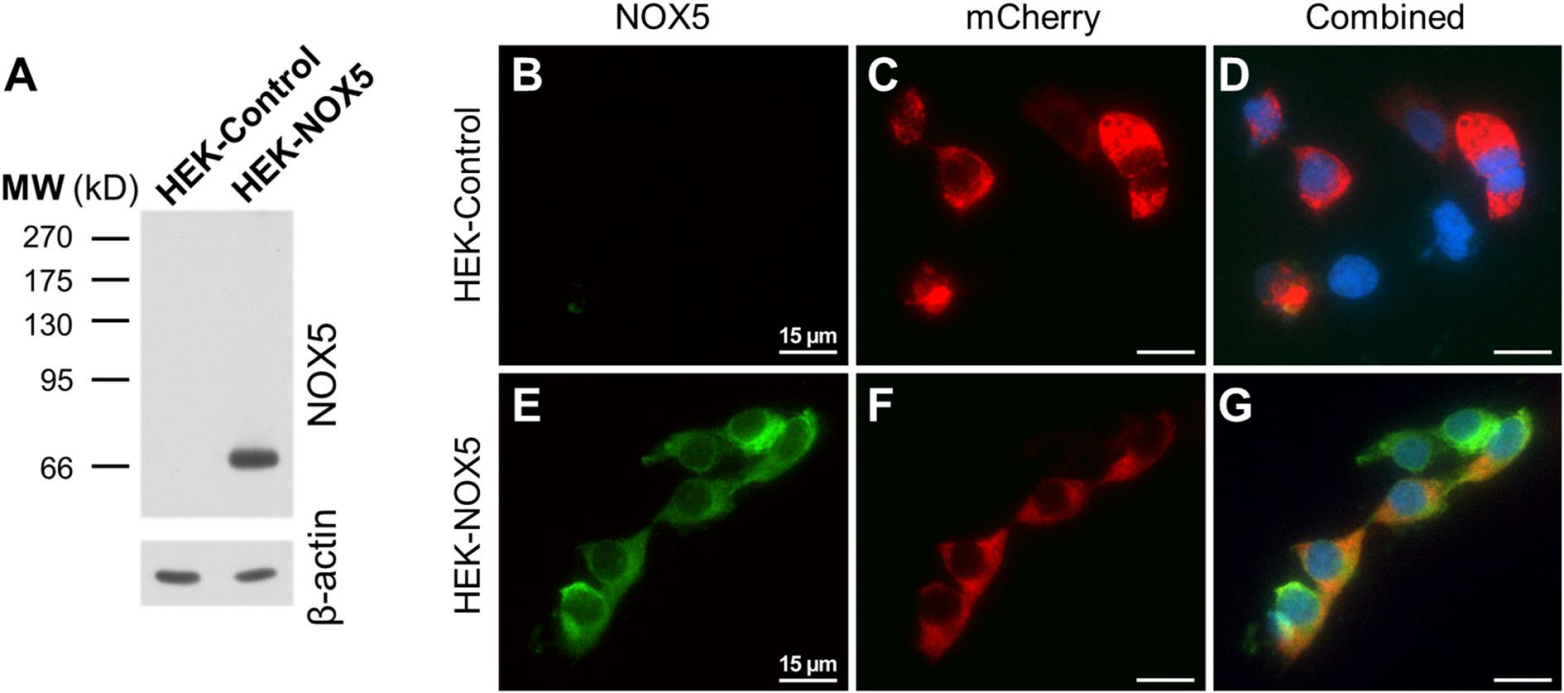
Detection of NOX5 protein in HEK293T cells. (**A**) Overexpressed NOX5 protein detection in the HEK293T cell lysate in Western blot. (**B**–**D**) Immunocytochemistry of control HEK293T cells. NOX5 staining (green, **B**), endoplasmic reticulum (ER)-targeted mCherry protein (red, **C**) and the merged signals along with nuclear staining (blue, **D**) are shown. (**E–G**) Immunocytochemistry of NOX5 overexpressing HEK293T cells. NOX5 staining (**E**) ER-targeted mCherry protein (**F**) and the merge of the two channels to check the extent of ER-localization of the NOX5 signal (**G**). Representative figures from 3 independent experiments.

In the next experiments, we used our novel monoclonal antibody to detect the NOX5 protein with Western blot. As shown in Fig. 2A a specific signal was only detected in cells that stably expressed NOX5 (Fig. 2A, Supplementary Fig. 1). We also evaluated the ability of the anti-NOX5 antibody to recognize NOX5 in immunocytochemistry staining. Figure 2B shows, that NOX5 was only present in transfected cells, where it mainly localized intracellularly. To assess whether the intracellular localization is due to its presence in the endoplasmic reticulum (ER), we co-expressed an ER-targeted mCherry protein, which showed highly similar cellular distribution pattern to that of NOX5 (Figs. 2B–G).

### Detection of the activity of endogenously expressed NOX5 in UACC-257 human melanoma cells

**Figure 3:**
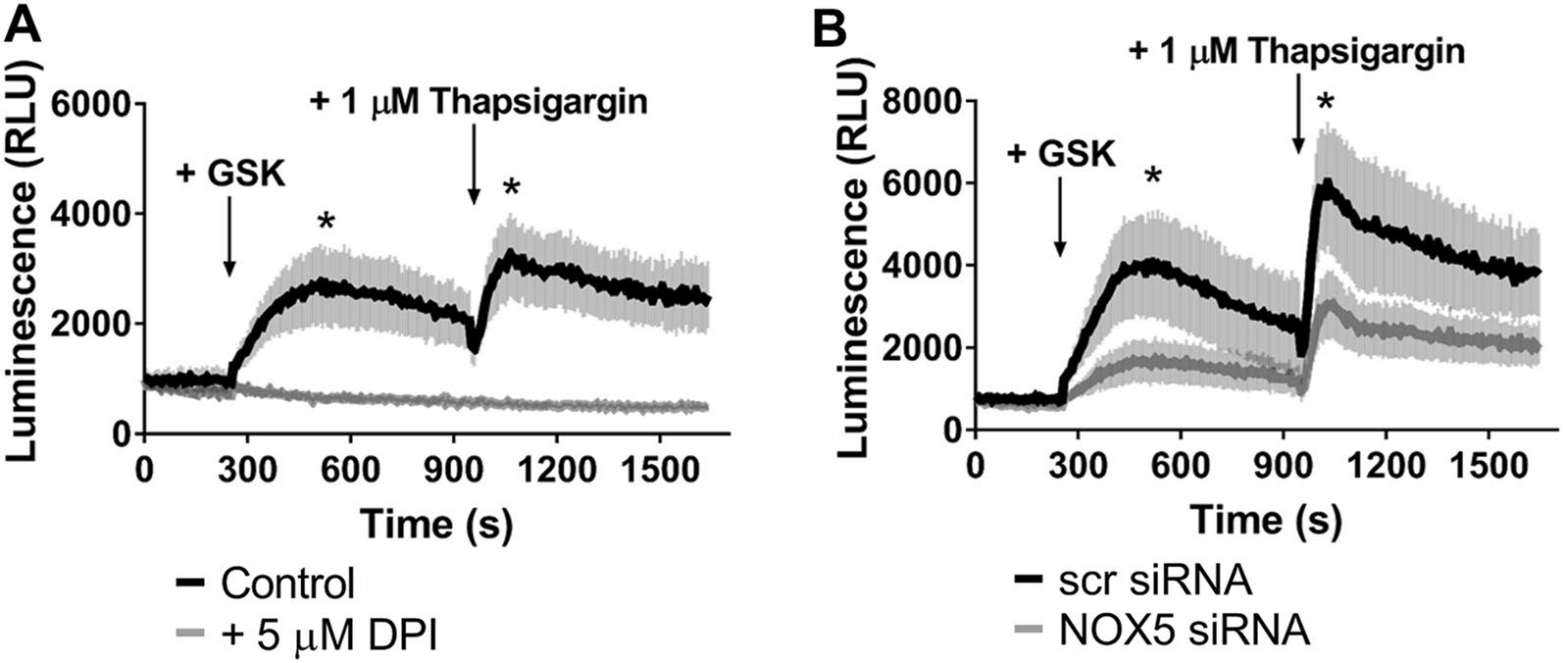
Detection of the endogenous NOX5 activity in UACC-257 cells. Cells were stimulated with the TRPV4 channel agonist GSK1016790A (GSK, at 1.5 or 2 nM final concentration) and the SERCA inhibitor thapsigargin. (**A**) Inhibition of the NOX5 enzyme by adding DPI at the starting of the measurement. The superoxide signal of the DPI treated cells was completely absent. *n* = 4 (**B**) The extracellular superoxide detection of scrambled (scr) and NOX5 specific siRNA transfected UACC-257 cells two days after transfection. The NOX5 silenced cells produce less extracellularly detectable superoxide. *n* = 6, mean ± SEM.

**Figure 4:**
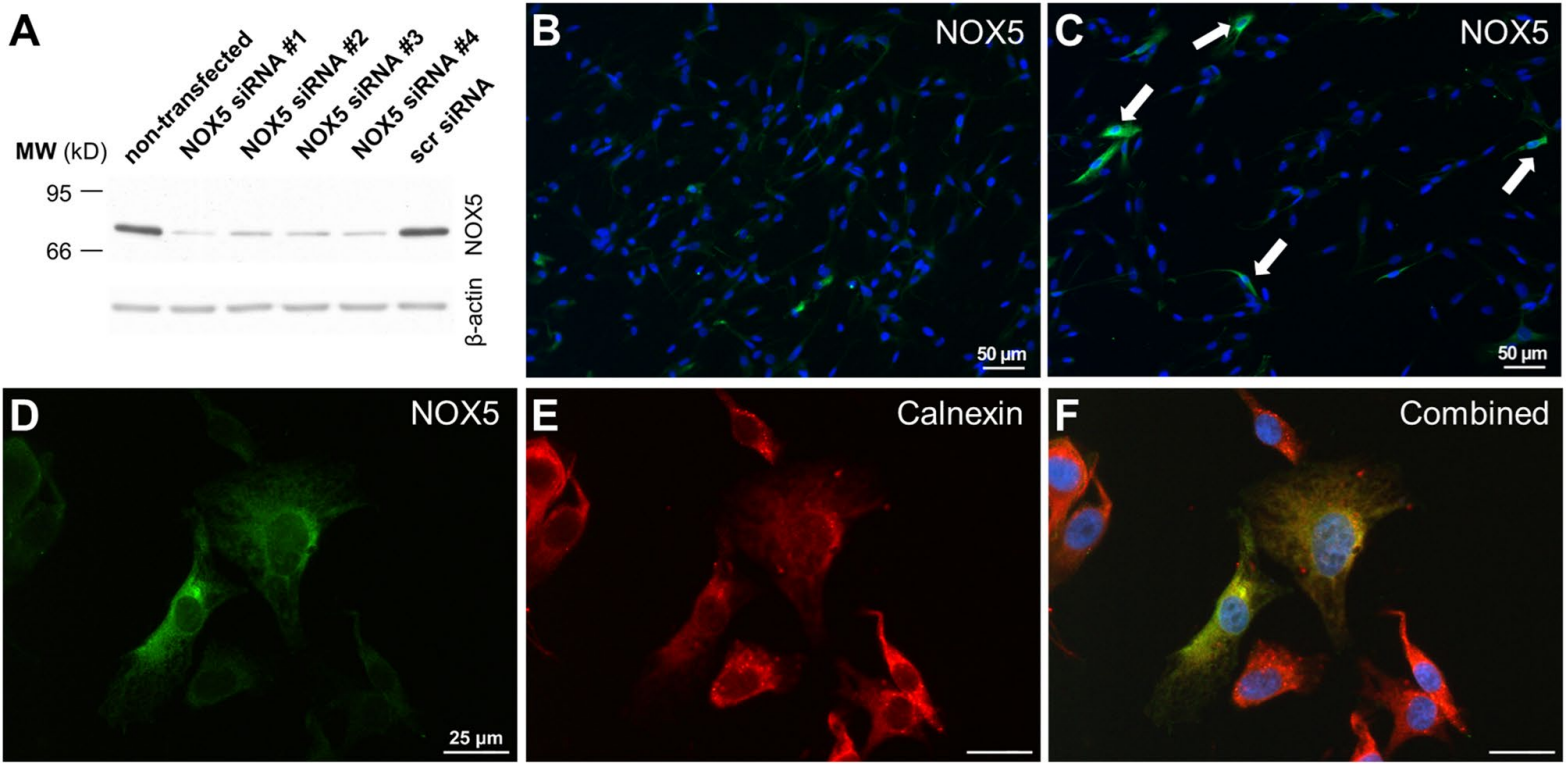
Detection of the endogenous NOX5 protein in the UACC-257 cells. (**A**) Validation of the novel NOX5 antibody on UACC-257 cells using different UACC-257 cell lysates. The NOX5 protein expression was successfully attenuated by four different NOX5 siRNAs (NOX5 siRNA #1-#4), while the NOX5 amount did not decrease in the scrambled (scr) siRNA transfected cells. (**B** and **C**) Immunocytochemistry of UACC-257 cells transfected with NOX5 siRNA (**B**) or scrambled siRNA (**C**). (**D**, **E**) UACC-257 cells co-immunostained for NOX5 (green, **D**) and for calnexin (red, **E**) show overlapping signals (combined, **F**), which localize mostly to the endoplasmic reticulum and the nuclear membrane. Cell nuclei are shown in blue. Representative figures from 2 independent Western blot experiments and 3 immunostainings.

Next, we set out to explore whether the anti-NOX5 antibody also recognizes the endogenously expressed NOX5 protein. To accomplish this, we chose to study the UACC-257 human melanoma cell line, which has previously been reported to express NOX5^13^. First we tested, whether UACC-257 cells produce superoxide in a NOX5-dependent way. As shown in Fig. 3, stimuli which elicit calcium signal, either by activating the store-operated calcium entry pathway (thapsigargin) or TRPV4 channels (GSK1016790A), increased superoxide production. The superoxide response was sensitive to DPI and suppression of NOX5 expression by siRNA treatment (Fig. 3A, B). We successfully detected NOX5 around 75 kD in UACC-257 cells in Western blot experiments (Fig. 4A, Supplementary Figs. 2 and 3), and the specificity of the signal was confirmed by reducing NOX5 levels with four different NOX5-targeting siRNAs (Fig. 4A). As shown in Supplementary Fig. 4 our antibody was already able to detect the NOX5 expression of as few as 1000 cells. Similar to the results of the experiments performed on NOX5-expressing HEK293T cells, we also successfully detected NOX5 in immunocytochemistry experiments in UACC-257 cells. Interestingly, we observed an uneven distribution of NOX5 expression among cells (Fig. 4B, C), suggesting that actual NOX5 levels may depend on the cell cycle. In NOX5-positive cells, we observed a substantial overlap in the subcellular distributions of NOX5 and the ER-marker calnexin (Fig. 4D–F).

### Detection of endogenously expressed NOX5 in human tissues by western blot and immunohistochemistry

**Figure 5:**
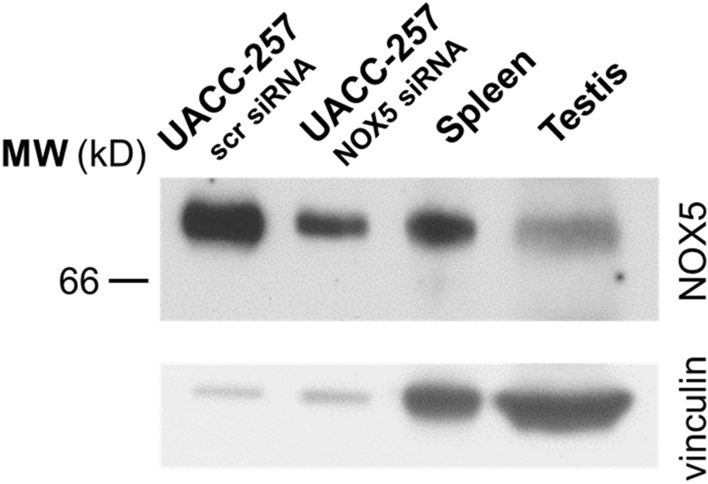
Western blot detection of the NOX5 protein in human spleen and testis tissue lysates. To verify the NOX5 specific signal, scrambled (scr) and NOX5 siRNA transfected UACC-257 cell lysates were tested alongside with the tissues. Representative figure from 3 independent experiments.

**Figure 6:**
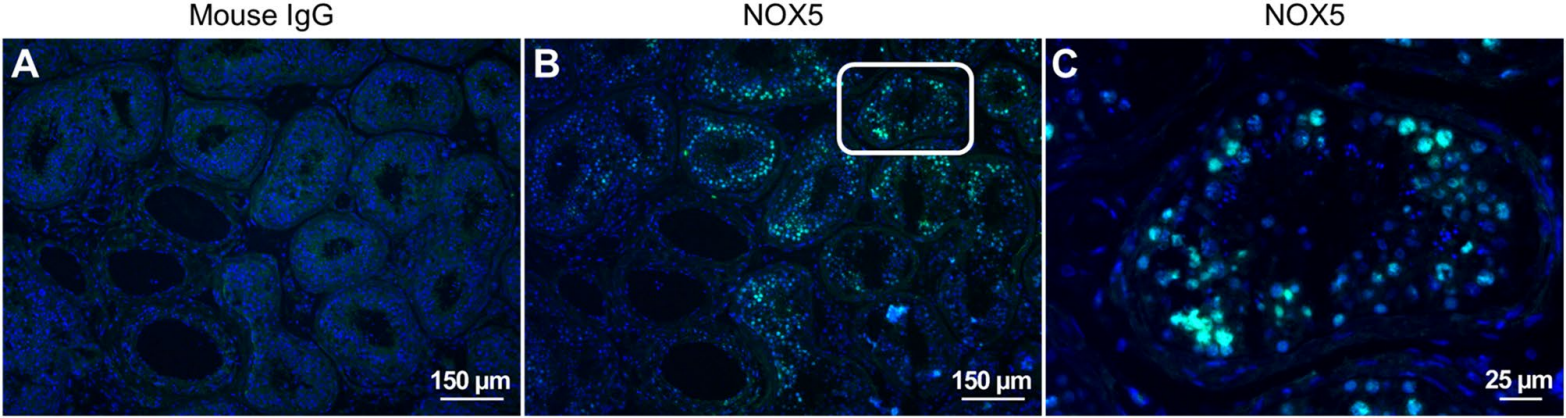
Immunohistochemical detection of NOX5 protein in tissue section of human testis The testis sample was either incubated with the control mouse IgG antibody (**A**), or with the novel NOX5 antibody (**B**), and the detected signals are shown in green. Nuclei are shown in blue. (**C**) The white-framed NOX5 stained testis region from (B) in a bigger magnification. The signals appear in the wall of seminiferous tubules, where NOX5 is associated with spermatocytes and spermatids. Representative figures from 2 independent experiments.

After confirming that our antibody can detect the endogenously expressed NOX5 protein, we attempted to detect NOX5 in human tissues where the enzyme is expressed at its highest levels. Western blot experiments were performed using protein lysates from spleen and testis and from UACC-257 cells as positive controls (Fig. 5, Supplementary Fig. 5). Figure 5 demonstrates that we could detect NOX5 protein in both tissues. Contrary to our expectations and literature data, we did not detect NOX5 in human spermatozoa (data not shown). In our next experiments we performed immunostaining on testis tissue sections. In these experiments immunostaining with non-specific mouse IgG served as negative control. In these experiments, we detected NOX5 positivity within the seminiferous tubules, where peripheral cells showed the highest NOX5 signal (Figs. 6A–C. Supplementary Fig. 6). Data from single-cell RNA experiments support the result of these immunostaining experiments as spermatogonia, spermatocytes and spermatids were identified to express NOX5 mRNA (https://www.proteinatlas.org/ENSG00000255346-NOX5/single+cell+type/testis).

### Detection of NOX5 in the human ovary

**Figure 7:**
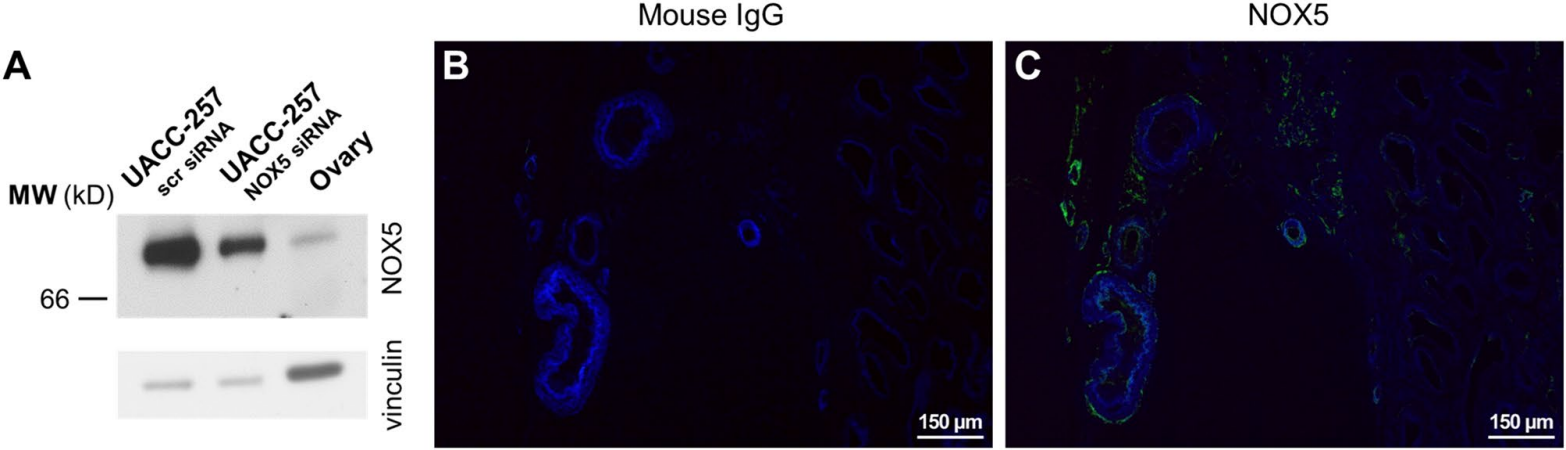
Detection of NOX5 protein in human ovary. (**A**) NOX5 protein detection in human ovary lysate in Western blot. To verify the NOX5 specific signal, scrambled (scr) and NOX5 siRNA transfected UACC-257 cell lysates were tested alongside with the tissues. Representative figure from 3 independent experiments. (**B**) Immunohistochemistry with the control mouse IgG antibody. (**C**) Immunohistochemistry with the novel NOX5 antibody. Signals form the antibodies are shown in green, while nuclei are shown in blue.

In silico analysis of NOX5 expression revealed that NOX5 is also expressed in the human ovary, where theca cells and fibroblasts contain/express NOX5 (https://www.proteinatlas.org/ENSG00000255346-NOX5/single+cell+type/ovary). As shown in Fig. 7A, we could detect the NOX5 protein in human ovarian lysate by Western blot (Fig. 7A and Supplementary Fig. 7). Immunostaining of human ovary tissue sections indicated that NOX5 is expressed in the interstitium of the ovaries. (Fig. 7B, C).

### Analysis of NOX5 content in human vascular cells

**Figure 8:**
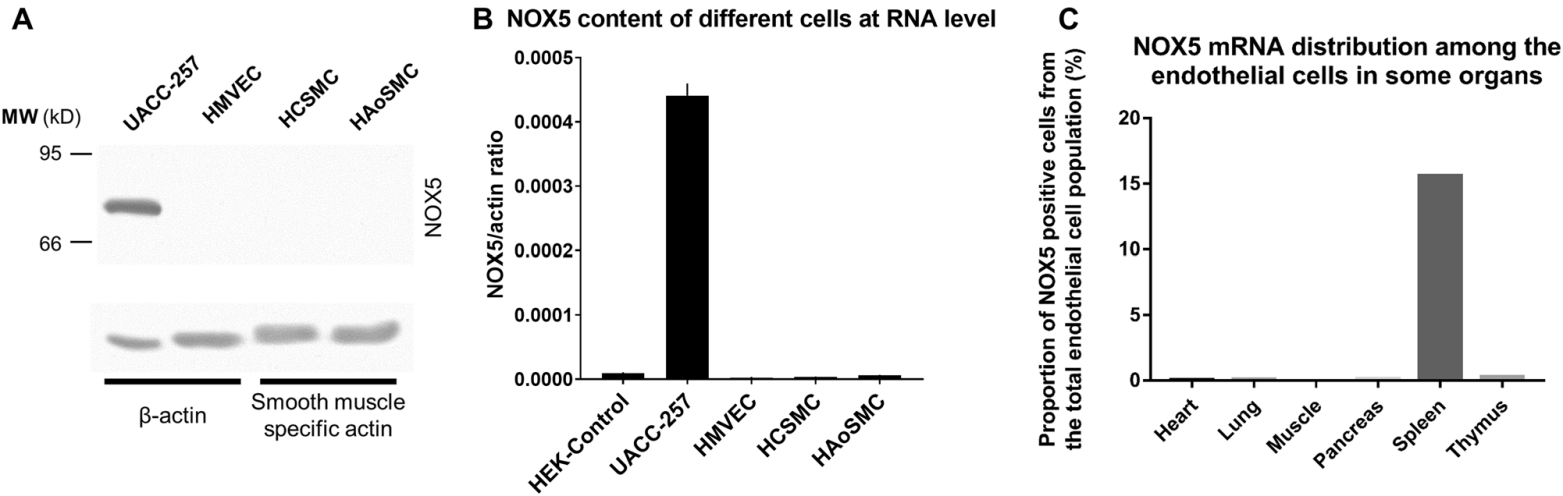
Detection of NOX5 protein and mRNA in human primary vascular cells and in endothelial cells from different tissues. Different labels refer to the following samples: HEK-Control: HEK293 cells transfected with an empty pSB/CMV/MCS/Puro vector; UACC-257 cells; HCSMC: Human coronary smooth muscle cells; HAoSMC: Human aortic smooth muscle cells; HMVEC: Human cardiac microvascular endothelial cells. (**A**) Detection of NOX5 protein in human primary vascular cells in Western blot. To verify the purity of the smooth muscle cells, an actin antibody specific for smooth muscle actin was applied. Representative figure from 2 independent experiments. (**B**) Detection of NOX5 mRNA in human primary vascular cells by qPCR. *n* = 2–3, mean ± SEM (**C**) Distribution of NOX5 mRNA among endothelial cells in some organs according to the Tabula Sapiens (multiple-organ, single-cell transcriptomic atlas of humans) database. The most NOX5 positive endothelial cells can be found in the spleen. The muscle column contains both the skeletal and smooth muscle tissues.

**Figure 9:**
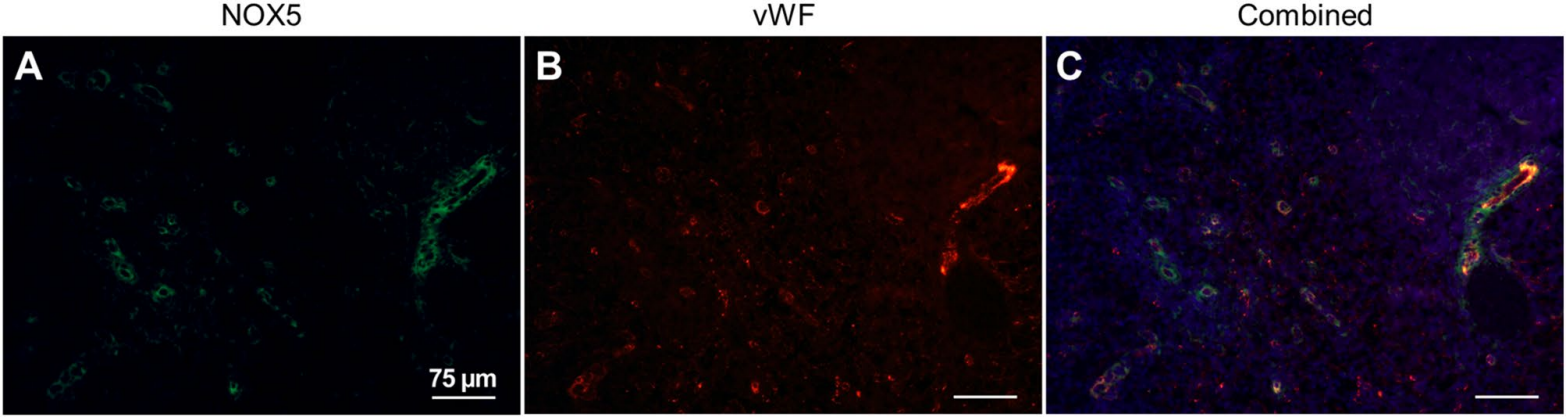
Immunohistochemical detection of NOX5 protein in tissue section of human spleen. (**A**) NOX5 labeling of the tissue is shown in green, while (**B**) the endothelial cell specific vWF labeling in red. (**C**) The red and green channels are merged and supplemented with the blue nuclear stain. Overlapping distribution of the intense red and green signals indicates significant NOX5 expression in splenic endothelial cells. Representative figures from 2 independent experiments.

Although numerous studies have addressed the possible function of NOX5 in the human vasculature^14–16^, our knowledge of the expression and localization of NOX5 at the protein level is still scarce. We, therefore, analyzed the NOX5 mRNA and protein expression in three different cell types: human coronary smooth muscle cells, human aortic smooth muscle cells and human cardiac microvascular endothelial cells (Figs. 8A, B, Supplementary Fig. 8). UACC-257 cells served as positive controls in these experiments. Our results suggest that NOX5 expression is very low in these primary cells. We were interested whether single-cell RNA sequencing results indicate the expression of NOX5 in vascular cell populations. To this end, we analyzed NOX5 expression in Tabula Sapiens, a multi-organ, single-cell transcriptomic atlas of nearly 500,000 human cells^17^. As shown in Fig. 8C, only endothelial cells of the spleen showed NOX5 expression, while no detectable NOX5 expression was observed in endothelial cells of other major organs (Fig. 8C). Importantly, we found no evidence for NOX5 expression in immune cell populations of the spleen (data not shown). In our next experiments, we performed immunostaining on human spleen sections, where we observed co-localization of the NOX5 protein with the endothelial cell marker von Willebrand factor (Fig. 9A–C). In accordance with the results of single-cell RNA sequencing experiments, we did not detect NOX5 in endothelial cells of skeletal muscle (Supplementary Fig. 9).

## Discussion

NOX5 is probably the most enigmatic member of the NADPH oxidase family^8^. The human NOX5 enzyme was identified more than 20 years ago, though its physiological function remains to be identified. Studies addressing the function of NOX5 are hampered by the fact that the NOX5 gene is absent in rodents including the two most frequently used model organisms: mice and rats. On the other hand, the details of the molecular level appearance of NOXes were first obtained from structural studies on NOX5^18^. NOX5 is also unique in that no additional membrane or cytosolic proteins are required for its activation, whereas the activity of all other NOX/DUOX isoforms depends on additional subunits. NOX5 is activated by an increase in cytosolic Ca^2+^ levels, mediated by calcium binding of EF-hand motifs located in the N-terminus of the protein^19^. Based on its structure and the mechanism of activation NOX5 highly resembles ancient respiratory burst oxidize homologs (Rboh proteins) which function in plants^7^. The expression pattern of NOX5 is also unique, as the mRNA of NOX5 was first described only in the spleen and testis^6,20^. However, subsequent studies have expanded the number of tissue and cell types where NOX5 was found. Detection of NOX5 expression in cells of the cardiovascular system attracted a lot of attention, since altered production of ROS has been implicated in the pathogenesis of various cardiovascular diseases^21^. It should be emphasized, however, that most research on NOX5 only detected the mRNA by PCR and few studies provided data on the actual protein level in the analyzed cells or tissues. This problem is not unique to NOX5 research, since the specific detection of non-phagocytic NOX isoforms still represents a major challenge to the NOX research field^11^.

In this work, we developed and characterized a novel monoclonal, NOX5-specific antibody and described the detection of the human NOX5 protein in normal human tissues for the first time. In these experiments, we could confirm the expression of NOX5 in the spleen and testis at the protein level. Immunohistochemical staining on human spleen tissue also confirmed the presence of NOX5, however, our result did not indicate the association of NOX5 expression with lymphocytes (T- and B-lymphocyte-rich areas) which was originally suggested by the in situ hybridization experiments of Banfi et al.^6^. Marzaioli et al*.*^22^ reported NOX5 expression in monocytes and dendritic cells, however single-cell RNA sequencing analysis of human spleen and blood immune cells did not indicate NOX5 mRNA expression in human lymphocytes or other leukocyte populations. Our immunostainig results, along with single-cell RNA sequencing data suggest that NOX5 expression in the spleen is of endothelial origin.

We also successfully detected NOX5 in human testis lysate, and immunostaining experiments revealed NOX5 protein expression in developing sperm precursor forms. To our knowledge, this is the first successful NOX5 immunostaining in the testis. Based on the results of the immunostaining experiments we cannot identify the exact developmental stage(s) in that NOX5 is present in the cells. However, our results are in agreement with the results of previous in situ hybridization experiments, where the NOX5 mRNA was detected^6^. Furthermore, single-cell RNA sequencing analysis also revealed the expression of NOX5 mRNA in spermatocytes and spermatids. These results suggest that NOX5 has an unidentified function during spermatogenesis. Several publications suggested that NOX5 is present in mature spermatozoa, although relatively few studies were performed on human cells. Most of the NOX5-related research work has accepted the assumption that ROS production by mature spermatozoa is due to NOX5 activity^23,24^. Reviewing of the NOX5-related publications revealed only two papers, where NOX5 was detected by Western blot at the protein level^25,26^, however, the same antibody was used in both works. In a different study, NOX5 was detected at the protein level in equine testis and spermatozoa, but the antibody also detected a NOX5 signal in several other tissues, where NOX5 mRNA is not expressed^27^. In our experiments, we did not detect NOX5 in spermatozoa, although several control experiments indicated that our anti-NOX5 antibody can detect the protein with high sensitivity. The results of our immunostaining experiments also make a case against the expression of NOX5 in spermatozoa, because we did not observe NOX5-positive cells in the center of seminiferous tubules, where spermatozoa are normally present in high amounts. On the other hand, we cannot exclude the possibility that NOX5 becomes part of a larger protein complex in spermatozoa, thus preventing the access of antibodies to the protein.

Another important finding of our paper is the detection of NOX5 protein in the human ovary. According to publicly available single-cell RNA sequencing data, theca cells, and fibroblasts express NOX5 in the human ovary. The results of our immunostaining experiments are in agreement with RNA sequencing data, as we detected NOX5 around developing follicles. The function of NOX5 in the female and male reproductive organs remains to be identified. It is interesting to note that NOX5 can transform calcium signals into a ROS response and such coupling has an essential role in the fertilization of lower species, such as sea urchins^28^. The role of ROS in the fertilization of vertebrates is less understood, and based on the expression pattern of NOX5, it seems unlikely that the enzyme produces ROS during fertilization.

NOX5 was first described in vascular cells by BelAiba et al.^15^ who detected NOX5 in both vascular endothelial and smooth muscle cells. Subsequently, other groups have also identified the enzyme in vascular cells^14^, and suggested that NOX5 contributes to the development of human coronary artery disease^29^. A pathogenic role for NOX5 in the cardiovascular system was also suggested by others^16^. However, it is important to note that in several works related to NOX5, the enzyme was detected only at the mRNA level by PCR, and in those cases where the protein was detected, detailed characterization of the antibodies was usually not performed. Using our anti-NOX5 antibody, the NOX5 protein content of human cardiac microvascular endothelial cells, and human coronary and aortic smooth muscle cells was under detection limit. QPCR analysis also indicated the lack of NOX5 in the analyzed primary cells. We also studied multiple single-cell RNA sequencing datasets and spleen was the only organ where NOX5 expression was detected in significant percentage of endothelial cells. The reason for the contradiction between already published results and our observations is not entirely clear, but certain stimuli may be necessary for the induction of NOX5 in vascular cells. Vasoactive agents, including angiotensin II, endothelin-1, and PDGF were all described to induce NOX5 expression in vascular cells. It should be also noted that our antibody was raised against the N-terminal 167 amino acids of human NOX5, therefore the short NOX5 isoform (NOX5ε or NOX5s) is not detected by our reagent. On the other hand, it would be also important to apply rigorous genetic methods to analyze NOX5 function in the pathogenesis of cardiovascular diseases. We have recently generated a NOX5-deficient rabbit line, where we observed a protective role of NOX5 against the development of atherosclerosis induced by cholesterol feeding^10^. Downregulation of NOX5 expression by hypermethylation was described as a possible pathogenic factor in congenital heart disease^30,31^, however, the underlying mechanisms still await clarification.

We believe that the development and characterization of a novel anti-NOX5 antibody will help to elucidate the hitherto unknown functions of NOX5.

## Methods

### Materials

All reagents and chemicals were purchased from Sigma-Aldrich unless otherwise specified. Human, adult, normal spleen (NB820-59259) and testis (NB820-59266) lysates and spleen (NBP2-30202), testis (NBP2-75940), skeletal muscle (NBP2-77813) and ovary (NBP2-30190) tissue slides were from Novus Biologicals. Human, adult, normal ovary lysate (HT-406-ZY) was from BioCat. The β-actin (A1978), the vinculin (V9131) and the α-smooth muscle actin (A5228) antibodies were from Sigma-Aldrich, the vWF polyclonal antibody (PA516634), the Alexa Fluor Plus 488 anti-mouse secondary antibody (A32766), the Alexa Fluor 568 anti-rabbit secondary antibody (A10042) and the HRP conjugated anti-mouse secondary antibody (31,432) were from Thermo Fischer Scientific. The mouse IgG1 antibody (554,121) was from BD Biosciences, the calnexin antibody (2679 T) was from Cell Signaling Technology.

### Cell culture

HEK 293T cells were obtained from ATCC (American Type Culture Collection). HEK293T cells were grown in Dulbecco's modified Eagle medium (DMEM) with 4.5 g/l glucose and glutamine (Lonza or Capricorn) supplemented with 10% heat-inactivated fetal bovine serum (Lonza), 50 U/ml penicillin and 50 μg/ml streptomycin (Lonza). UACC-257 cells were grown in Roswell Park Memorial Institute (RPMI) 1640 medium (Lonza) with the same supplements. Human coronary smooth muscle cells (HCSMC) and human aortic smooth muscle cells (HAoSMC) were cultured in smooth muscle cell growth medium (PromoCell) supplemented with 50 µl/ml fetal calf serum, 0.5 ng/ml epidermal growth factor, 2 ng/ml basic fibroblast growth factor and 5 μg/ml insulin. Human cardiac microvascular endothelial cells were grown in EGM-2 endothelial cell growth medium-2 BulletKit (Lonza). All cell lines were kept in a humidified incubator with an atmosphere of 5% CO_2_ at 37 °C.

### Cloning

Open reading frame of the human NOX5 cDNA (BC125098.1) was inserted into pcDNA3.1/V5-His-TOPO vector with TA cloning strategy (Invitrogen, Life Technologies, Waltham, MA, USA). The NOX5 coding sequence was amplified from this plasmid by PCR with Phusion Hot Start DNA Polymerase (Thermo Fischer Scientific) with an extra 5’ EcoRV and 3’ XbaI site. Both the insert and the pSB/CMV/MCS/Puro vector were digested with the EcoRV and XbaI restriction enzymes (Thermo Fischer Scientific), then purified with the MEGAquick-spin Plus Total Fragment DNA Purification Kit (Intron Biotechnology). Finally, the digested DNA-s were ligated by a T4 DNA ligase (Thermo Fischer Scientific) then the DNA was transformed into TOP10 *E. coli* bacteria (Thermo Fischer Scientific). The plasmid was purified with GeneJET Plasmid Miniprep Kit (Thermo Fischer Scientific).

To create the GST-NOX5 construct, the first 167 amino acids of the human NOX5 was amplified from the original plasmid with Phusion Hot Start DNA Polymerase (Thermo Fischer Scientific). Both the insert and the pGEX-4 T-1 vector were digested with the BamHI and XhoI enzymes, then purified and ligated as described above. Finally, the DNA was transformed into TOP 10 *E. coli* bacteria, and after the plasmid preparation, the constructs were checked with restriction digestion.

To create the NOX5-His_6_ construct, cDNA encoding the first 167 amino acids of the human NOX5 was amplified from the original plasmid with Phusion Hot Start DNA Polymerase (Thermo Fischer Scientific). To reach the poly-His-tagged NOX5, the aLICator LIC Cloning and Expression Kit was used (Thermo Fischer Scientific) according to the description of the product.

In all cloning experiments, the final sequence was verified with sequencing (Microsynth AG, Balgach, Switzerland).

### siRNA and DNA transfection

UACC-257 cells (ATCC) were seeded to 6- and 24-well plates. To silence NOX5, 4 different siRNA (ON-TARGETplus Human NOX5 siRNA: J-010195-06-0002, J-010195-07-0002, J-010195-08-0002, J-010195-09-0002, Horizon) and 1 control siRNA (D-001210-02-20, Thermo Scientific) were applied in a final concentration of 40 nM. The cells were transfected in suspension with Lipofectamine RNAiMAX reagent (Thermo Fischer Scientific) and then cultured for 2 days before processing.

HEK293T cells (ATCC) were seeded to a 6-well plate and were transfected with Lipofectamine LTX & PLUS reagent (Thermo Fischer Scientific) in suspension. To create stable cell lines, the cells were co-transfected with transposase enzyme coding plasmid and an IR/DR sequence containing plasmid in a ratio of 1:5, with a total of 2 µg of DNA. Two days after transfection, the resistant clones were selected in the presence of 7.5 µg/ml puromycin (A1113803, Thermo Fischer Scientific) for 2 weeks. The ER-targeted mCherry construct^32^ was transfected the same way (500 ng DNA/well in a 24-well plate).

### RNA preparation, cDNA synthesis and quantitative PCR (qPCR)

Total RNA was prepared using the NucleoSpin RNA, Mini kit for RNA purification (Macherey–Nagel) according to the manufacturer's recommendation. The purification also included on-column DNA digestion with DNase I enzyme. The concentration of the isolated RNA was measured by a NanoDrop One system (Thermo Fischer Scientific). The High-Capacity cDNA Reverse Transcription Kit (Thermo Fischer Scientific) was used for cDNA synthesis, following the recommended protocol of the manufacturer. The cDNA synthesis was started from 2 µg RNA and supplemented with 1 µl Ribolock RNase inhibitor (Thermo Fischer Scientific). For qPCR the LightCycler 480 Probes Master (Roche) ready-to-use reaction mix was applied. The reaction was performed in a final volume of 10 µl, combining 0.5 µl of the probe, 0.5 µl of cDNA, 5 µl of Probe Master, and 4 µl of water. The measurements were performed by LightCycler 480 System (Roche). The following Taqman probes were used: NOX5: Hs00225846_m1, Actin: Hs00357333_g1. The NOX5 Taqman probe detects all known splice variants of Nox5.

### Western blot

After washing with PBS (Phosphate buffered saline), cells were harvested with RIPA solution (150 mM NaCl, 1% Triton X-100, 1% Na-deoxycholate, 0,1% SDS, 20 mM Tris–Cl, 1 mM EDTA, 1 mM EGTA, pH = 8) prepared with protease inhibitors: 1 mM PMSF and 1 pill cOmplete Mini EDTA-free Protease Inhibitor (Roche) per 10 ml RIPA solution. The samples were left on ice for 10 min then the detergent-insoluble fraction was removed by centrifugation at 16,100 × g for 10 min at 4 °C. Supernatants were mixed with Laemmli sample buffer containing 5% 2-mercaptoethanol. Samples were separated on 10% SDS polyacrylamide gels and blotted onto nitrocellulose membrane. The membranes were blocked using 5% skimmed milk powder in 0.1% Tween-20 containing PBS (PBS-Tween). The primary antibody was applied in PBS-Tween containing 5% BSA for at least 1 h at ambient temperature (5–10 µg/ml NOX5 Ab, 1:500 α-smooth muscle actin Ab, 1:5000 β-actin and vinculin Ab), followed by washing the membrane 6 × with PBS-Tween for 30 min in total. The secondary antibody was dissolved in the blocking buffer (1:10,000) and incubated with the membrane for 1 h. Finally, the membrane was washed again as indicated above, then Westernbright ECL kit (Advansta) reagents were added to the membrane. The luminescence signal was detected using Fuji Medical X-ray films (Fujifilm). The films were scanned with Canon CanoScan 8800F at 600 dpi. The results were analyzed using the ImageJ software (http://imagej.org).

### Immunocytochemistry

The cells were washed once with ice-cold PBS and then fixed with 4% PFA in PBS for 20 min on ice. Samples were washed 6 × with ice-cold PBS, then incubated for 10 min with 10 mM glycine solution. Samples were washed 2 × with 10 mM glycine solution, then 2 × with PBS, after which the cells were permeabilized with 0.1% Triton X-100 in PBS with 1% BSA for 20 min. Next, the samples were blocked for at least 1 h with 5% BSA in PBS at room temperature. The primary antibody was diluted in the blocking buffer (5 µg/ml NOX5 Ab, 1:100 Calnexin Ab) and left on the cells overnight at 4 °C in a humidified chamber followed by washing 8 × with PBS on the next day. The secondary antibody (1:1000) and the nuclear staining (4 µM Höechst 33342) were applied in the blocking buffer for 1 h at room temperature in the dark. Finally, the samples were washed 6–8 × with PBS and covered with a coverslip using Mowiol^®^. The results were imaged with a Leica DMI6000B fluorescence microscope (Leica Microsystems). All contrast adjustments were carried out the same way of the control and NOX5 staining.

### Immunohistochemistry

For deparaffinization and rehydration, slides were placed in a 60 °C thermoblock for 2 h. Then the samples were sequentially immersed in xylene for 2 × 10 min, followed by 2 min in absolute, 96%, 85%, 70%, 60%, and 50% ethanol. Finally, slides were washed in running tap water and then in distilled water for 1–1 min.

For antigen retrieval, samples were bathed in 10 mM Na-citrate solution (pH = 6) in a pressure-cooker for 20 min. After washing with PBS, ready-to-use pepsin solution was added to the tissues for 10 min at 37 °C. Finally, the slides were washed with PBS.

Immunostaining was performed with Alexa Fluor 488 Tyramide SuperBoost Kit (ThermoFisher). To inhibit endogen peroxidases, tissue samples were incubated with 3% H_2_O_2_ solution for 1 h at ambient temperature, washed 3 × with PBS, then blocked with 10% normal goat serum and 0.5% TritonX-100 containing PBS for 1 h at ambient temperature. The primary antibody (1:100 vWF Ab, 25 µg/ml NOX5 Ab and mouse IgG Ab, except for the spleen, where 7.5 µg/ml was used) was applied in the manufacturer's blocking buffer supplemented with 0.1% TritonX-100, and samples were incubated overnight at 4 °C in a humidified chamber. The slides were washed with PBS 6 × for 30 min in total on an orbital shaker. Samples were incubated with the secondary antibody and nuclear staining (1:500 anti-rabbit 568 Ab, 4 µM Höechst 33342) for 1 h at room temperature in the dark, then washed as before. Tyramide working reagent was prepared and applied on the samples for 10 min. Finally, STOP reagent was added to the sections, and the slides were washed 5 × with PBS and covered with a coverslip using Mowiol^®^. The results were recorded with the same microscope as indicated before. All contrast adjustments were also carried out the same way for the control and NOX5 staining.

### Superoxide and Ca^2+^ measurements

All measurements were performed with a CLARIOstar Plus Microplate Reader (BMG LABTECH) at 37 °C. Black chimney, clear 96-well plates (Greiner 655090) were used for Ca^2+^ measurements, and white chimney, solid bottom, 96-well plates (Greiner 655083) were used for superoxide measurements. The stimuli were injected or pipetted into the wells, and the final volume was 100 μl/well in all cases.

Superoxide measurements were performed using circa 40,000 cells/well in suspension in a 1:1 mixture of H-medium (145 mM NaCl, 5 mM KCl, 1 mM MgCl_2_, 0.8 mM CaCl_2_, 5 mM glucose, 10 mM HEPES) and Diogenes reagent (National Diagnostics). The extracellular superoxide signal was detected in luminescence mode.

Ca^2+^ measurements were performed on confluent, adherent cell cultures in H-medium. The cells were loaded with 2 μM Fura-2 AM dye (Life Technologies) for 30 min at 37 °C in the dark, then washed with H-medium. Fura-2 was excited at 335 and 380 nm, and respective emission signals were detected at 510 nm, and their ratio (335/380) was analyzed.

### Expression of fused proteins

The DNA constructs encoding GST-NOX5 and NOX5-His_6_ were transformed into the chemically competent BL21 *E. Coli* strain (Thermo Fischer Scientific). The bacteria were seeded on agar plates and incubated overnight at 37 °C. 1–1 colony was inoculated into 100–100 ml of Terrific Broth medium (1.2% tryptone, 2.4% yeast extract, 0.5% glycerol, 17 mM KH_2_PO_4_, 72 mM K_2_HPO_4_, 50 µg/ml ampicillin) and the cultures were grown overnight in an orbital shaker at 37 °C at 180 rpm. 900–1400 ml Terrific Broth medium was added to the bacterial suspensions, and at an OD of 0.5–0.6 the protein production was induced by adding IPTG (Thermo Fischer Scientific), in a final concentration of 0.3–0.5 mM for 3–4 h.

### Purification of GST-NOX5 protein

The bacteria were centrifuged for 15 min, at 4 °C and 5600 g. The pellets were suspended in 10 ml lysis buffer (50 mM Tris, 50 mM NaCl, 5 mM MgCl_2_, 2 mM DTT, 2 mM PMSF, 1 pill cOmplete Mini EDTA-free Protease Inhibitor (Roche), pH = 7,6) on ice, then sonicated 5 × for 1 min on ice. The insoluble fraction was removed by centrifugation for 20 min, at 4 °C and 20,000 g. Glutathione beads (Pierce Glutathione Superflow Agarose, Thermo Fischer Scientific) were added to the supernatant and incubated 1 h at 4 °C on a rotator. The beads were washed with cold lysis buffer, then the recombinant protein was eluted with 2.5 ml of glutathione elution buffer (5 mM reduced glutathione, 50 mM Tris, 151 mM NaCl, 5 mM MgCl_2_, 1 mM DTT) at 4 °C, on a rotator, for 30 min. Finally, the protein solution was dialyzed against 5 L of PBS overnight at 4 °C.

### Purification of NOX5-His_6_ protein

Bacterial pellets were produced the same way as in case of GST-NOX5 protein. However, in this case, a phosphate-based lysis buffer was used (50 mM NaH_2_PO_4_, 300 mM NaCl, 2 mM PMSF, 1 pill cOmplete Mini EDTA-free Protease Inhibitor, pH = 8), and Nickel-beads (HIS-Select HF Nickel Affinity Gel) was used to extract the recombinant protein. The lysis buffer was incubated with the beads overnight at 4 °C on a rotator. Beads were washed 5 × with 5–5 ml phosphate lysis buffer containing 4 mM imidazole, and then proteins were eluted with phosphate buffer containing 500 mM imidazole, overnight at 4 °C on a rotator. Finally, the protein solution was dialyzed in PBS overnight at 4 °C.

### Animals

Hemizygous transgenic mice were used for the immunization experiments. These transgenic animals carry 5 copies of the bFcRn α-chain encoding gene (bovine FCGRT) in addition to the endogenous mouse FCGRT gene on BALB/c genetic background [BALB/c_Tg5_Bfcgrt]^12^. Mice were kept under specified pathogen-free (SPF) conditions in individual ventilation cages (IVC) in the animal house of the Department of Immunology, Eötvös Loránd University, Budapest, Hungary.

### Mouse immunization

7–8 weeks old female Tg mice were intraperitoneally (i.p.) immunized with 50 µg GST-NOX5 in complete Freund adjuvant (CFA) (1:1) and boosted 14, 28, and 42 days later with 25 µg GST-NOX5 in incomplete Freund adjuvant (IFA) (1:1). The sera of the animals were tested on day 56 by enzyme-linked immunosorbent assays (ELISA) using NOX5-His_6_ as antigen, and the best-responding animal was chosen for hybridoma and monoclonal antibody production.

### Hybridoma production and ELISA screening of the supernatants

Three days before fusion, the mouse was immunized with a final boost (25 µg GST-NOX5 in IFA). After harvesting the mouse splenocytes, the cells were used for fusion with SP2/0-Ag14 mouse myeloma cells (for a detailed description of the process see^33^. The hybridoma supernatants were tested by ELISA. Briefly, high-binding microplates (Greiner bio-one) were coated with NOX5-His_6_ solution in 0.1 M sodium carbonate-bicarbonate buffer (pH 9.6) for 2 h at room temperature and were washed with 0.1 M PBS-Tween (0.05% Tween20, pH 7.4, 5x). Concentrated supernatant samples were added to the wells and incubated for 1 h at room temperature. After washing (PBS-Tween, 5x), horseradish peroxidase (HRP)-labeled goat anti-mouse IgG was used (in 1:5000 dilution, Southern Biotechnology) as the secondary antibody. After washing 5 × with PBS-Tween, 3,3′,5,5′—tetramethylbenzidine (TMB) was used as the substrate for peroxidase activity detection, and optical density at 450 nm was measured using Multiskan SkyHigh Microplate Spectrophotometer (Thermo Fisher Scientific). Clones that appeared positive were further evaluated, and the NOX5 reactivity was confirmed by independent methods (Western blot, immunostainings) as well. A similar protocol was used for testing sera from mice, but the samples were serially diluted (5 × serial dilution from 100 × diluted sample) in the second step.

### Analysis of single-cell RNA sequencing data

Data from the Tabula Sapiens single-cell RNA sequencing dataset^17^ was analyzed using the Talk2Data platform (Bioturing Inc.). Single-cell RNA expression data was also analyzed in the Protein Atlas (www.proteinatlas.org).

### Statistics

Statistical analysis was performed using the Mann–Whitney U test. In experiments where calcium signal or superoxide production in cell lines was investigated, the measured values obtained in the same passages were normalized to the maximum detected value (ratio or RLU, respectively) and the peak responses to each stimulus were compared. The GraphPad Prism 7.0 program was used for the analysis.

### Ethics declaration

Experiments on mice were carried out in strict accordance with the recommendations of the Guide of the Institutional Animal Care and Ethics Committee at Eötvös Loránd University. The study was approved by the ethics committee of the Food Chain Safety and Animal Health Directorate of the Government Office of Pest County, Hungary (permission number: PEI/001/2196-2/2013). This study is reported in accordance with ARRIVE guidelines.

### Supplementary Information

Supplementary Figures.

## Data Availability

Data of this study are available upon reasonable request from the corresponding author.

## References

[1] Sies H, Belousov VV, Chandel NS, Davies MJ, Jones DP, Mann GE, Murphy MP, Yamamoto M, Winterbourn C (2022). Defining roles of specific reactive oxygen species (ROS) in cell biology and physiology. Nat. Rev. Mol. Cell Biol..

[2] Bedard K, Krause KH (2007). The NOX family of ROS-generating NADPH oxidases: physiology and pathophysiology. Physiol. Rev..

[3] Lambeth JD, Neish AS (2014). NOX enzymes and new thinking on reactive oxygen: A double-edged sword revisited. Annu. Rev. Pathol..

[4] Vermot A, Petit-Härtlein I, Smith SME, Fieschi F (2021). NADPH oxidases (NOX): An overview from discovery, molecular mechanisms to physiology and pathology. Antioxidants.

[5] Sirokmány G, Donkó Á, Geiszt M (2016). Nox/DUOX family of NADPH oxidases: Lessons from knockout mouse models. Trends Pharmacol. Sci..

[6] Bánfi B, Molnár G, Maturana A, Steger K, Hegedûs B, Demaurex N, Krause KH (2001). A Ca(2+)-activated NADPH oxidase in testis, spleen, and lymph nodes. J. Biol. Chem..

[7] Sumimoto H (2008). Structure, regulation and evolution of NOX-family NADPH oxidases that produce reactive oxygen species. FEBS J..

[8] Touyz RM, Anagnostopoulou A, Rios F, Montezano AC, Camargo LL (2019). NOX5: Molecular biology and pathophysiology. Exp. Physiol..

[9] Chen F, Yin C, Dimitropoulou C, Fulton DJ (2016). Cloning, characteristics, and functional analysis of rabbit NADPH oxidase 5. Front. Physiol..

[10] Petheő GL, Kerekes A, Mihálffy M, Donkó Á, Bodrogi L, Skoda G, Baráth M, Hoffmann OI, Szeles Z, Balázs B, Sirokmány G, Fábián JR, Tóth ZE, Baksa I, Kacskovics I, Hunyady L, Hiripi L, Bősze Z, Geiszt M (2021). Disruption of the NOX5 gene aggravates atherosclerosis in rabbits. Circ. Res..

[11] Diebold BA, Wilder SG, De Deken X, Meitzler JL, Doroshow JH, McCoy JW, Zhu Y, Lambeth JD (2019). Guidelines for the detection of NADPH oxidases by immunoblot and RT-qPCR. Methods Mol. Biol..

[12] Cervenak J, Bender B, Schneider Z, Magna M, Carstea BV, Liliom K, Erdei A, Bosze Z, Kacskovics I (2011). Neonatal FcR overexpression boosts humoral immune response in transgenic mice. J. Immunol..

[13] Antony S, Jiang G, Wu Y, Meitzler JL, Makhlouf HR, Haines DC, Butcher D, Hoon DS, Ji J, Zhang Y, Juhasz A, Lu J, Liu H, Dahan I, Konate M, Roy KK, Doroshow JH (2017). NADPH oxidase 5 (NOX5)-induced reactive oxygen signaling modulates normoxic HIF-1α and p27(Kip1) expression in malignant melanoma and other human tumors. Mol. Carcinog..

[14] Montezano AC, Burger D, Paravicini TM, Chignalia AZ, Yusuf H, Almasri M, He Y, Callera GE, He G, Krause KH, Lambeth D, Quinn MT, Touyz RM (2010). Nicotinamide adenine dinucleotide phosphate reduced oxidase 5 (NOX5) regulation by angiotensin II and endothelin-1 is mediated via calcium/calmodulin-dependent, rac-1-independent pathways in human endothelial cells. Circ. Res..

[15] BelAiba RS, Djordjevic T, Petry A, Diemer K, Bonello S, Banfi B, Hess J, Pogrebniak A, Bickel C, Görlach A (2007). NOX5 variants are functionally active in endothelial cells. Free Radic. Biol. Med..

[16] Camargo LL, Montezano AC, Hussain M, Wang Y, Zou Z, Rios FJ, Neves KB, Alves-Lopes R, Awan FR, Guzik TJ, Jensen T, Hartley RC, Touyz RM (2022). Central role of c-Src in NOX5-mediated redox signalling in vascular smooth muscle cells in human hypertension. Cardiovasc. Res..

[17] Jones RC, Karkanias J, Krasnow MA, Pisco AO, Quake SR, Salzman J, Yosef N, Bulthaup B, Brown P, Harper W, Hemenez M, Ponnusamy R, Salehi A, Sanagavarapu BA, Spallino E, Aaron KA, Concepcion W, Gardner JM, Kelly B, Neidlinger N, Wang Z, Crasta S, Kolluru S, Morri M, Pisco AO, Tan SY, Travaglini KJ, Xu C, Alcántara-Hernández M, Almanzar N, Antony J, Beyersdorf B, Burhan D, Calcuttawala K, Carter MM, Chan CKF, Chang CA, Chang S, Colville A, Crasta S, Culver RN, Cvijović I, D'Amato G, Ezran C, Galdos FX, Gillich A, Goodyer WR, Hang Y, Hayashi A, Houshdaran S, Huang X, Irwin JC, Jang S, Juanico JV, Kershner AM, Kim S, Kiss B, Kolluru S, Kong W, Kumar ME, Kuo AH, Leylek R, Li B, Loeb GB, Lu WJ, Mantri S, Markovic M, McAlpine PL, de Morree A, Morri M, Mrouj K, Mukherjee S, Muser T, Neuhöfer P, Nguyen TD, Perez K, Phansalkar R, Pisco AO, Puluca N, Qi Z, Rao P, Raquer-McKay H, Schaum N, Scott B, Seddighzadeh B, Segal J, Sen S, Sikandar S, Spencer SP, Steffes LC, Subramaniam VR, Swarup A, Swift M, Travaglini KJ, Van Treuren W, Trimm E, Veizades S, Vijayakumar S, Vo KC, Vorperian SK, Wang W, Weinstein HNW, Winkler J, Wu TTH, Xie J, Yung AR, Zhang Y, Detweiler AM, Mekonen H, Neff NF, Sit RV, Tan M, Yan J, Bean GR, Charu V, Forgó E, Martin BA, Ozawa MG, Silva O, Tan SY, Toland A, Vemuri VNP, Afik S, Awayan K, Botvinnik OB, Byrne A, Chen M, Dehghannasiri R, Detweiler AM, Gayoso A, Granados AA, Li Q, Mahmoudabadi G, McGeever A, de Morree A, Olivieri JE, Park M, Pisco AO, Ravikumar N, Salzman J, Stanley G, Swift M, Tan M, Tan W, Tarashansky AJ, Vanheusden R, Vorperian SK, Wang P, Wang S, Xing G, Xu C, Yosef N, Alcántara-Hernández M, Antony J, Chan CKF, Chang CA, Colville A, Crasta S, Culver R, Dethlefsen L, Ezran C, Gillich A, Hang Y, Ho PY, Irwin JC, Jang S, Kershner AM, Kong W, Kumar ME, Kuo AH, Leylek R, Liu S, Loeb GB, Lu WJ, Maltzman JS, Metzger RJ, de Morree A, Neuhöfer P, Perez K, Phansalkar R, Qi Z, Rao P, Raquer-McKay H, Sasagawa K, Scott B, Sinha R, Song H, Spencer SP, Swarup A, Swift M, Travaglini KJ, Trimm E, Veizades S, Vijayakumar S, Wang B, Wang W, Winkler J, Xie J, Yung AR, Artandi SE, Beachy PA, Clarke MF, Giudice LC, Huang FW, Huang KC, Idoyaga J, Kim SK, Krasnow M, Kuo CS, Nguyen P, Quake SR, Rando TA, Red-Horse K, Reiter J, Relman DA, Sonnenburg JL, Wang B, Wu A, Wu SM, Wyss-Coray T (2022). The tabula sapiens: A multiple-organ, single-cell transcriptomic atlas of humans. Science.

[18] Magnani F, Nenci S, Millana Fananas E, Ceccon M, Romero E, Fraaije MW, Mattevi A (2017). Crystal structures and atomic model of NADPH oxidase. Proc. Natl. Acad. Sci. USA.

[19] Millana Fañanás E, Todesca S, Sicorello A, Masino L, Pompach P, Magnani F, Pastore A, Mattevi A (2020). On the mechanism of calcium-dependent activation of NADPH oxidase 5 (NOX5). FEBS J..

[20] Cheng G, Cao Z, Xu X, van Meir EG, Lambeth JD (2001). Homologs of gp91phox: Cloning and tissue expression of NOX3, NOX4, and NOX5. Gene.

[21] Lassègue B, San Martín A, Griendling KK (2012). Biochemistry, physiology, and pathophysiology of NADPH oxidases in the cardiovascular system. Circ. Res..

[22] Marzaioli V, Hurtado-Nedelec M, Pintard C, Tlili A, Marie JC, Monteiro RC, Gougerot-Pocidalo MA, Dang PM, El-Benna J (2017). NOX5 and p22phox are 2 novel regulators of human monocytic differentiation into dendritic cells. Blood.

[23] Reyes-San-Martin C, Hamoh T, Zhang Y, Berendse L, Klijn C, Li R, Llumbet AE, Sigaeva A, Kawałko J, Mzyk A, Schirhagl R (2022). Nanoscale MRI for selective labeling and localized free radical measurements in the acrosomes of single sperm cells. ACS Nano.

[24] Keshtgar S, Ghani E (2022). Impact of calcium and reactive oxygen species on human sperm function: Role of NOX5. Andrologia.

[25] Musset B, Clark RA, DeCoursey TE, Petheo GL, Geiszt M, Chen Y, Cornell JE, Eddy CA, Brzyski RG, El Jamali A (2012). NOX5 in human spermatozoa: Expression, function, and regulation. J. Biol. Chem..

[26] Vatannejad A, Tavilani H, Sadeghi MR, Karimi M, Lakpour N, Amanpour S, Shabani Nashtaei M, Doosti M (2019). Evaluation of the NOX5 protein expression and oxidative stress in sperm from asthenozoospermic men compared to normozoospermic men. J. Endocrinol. Invest..

[27] Sabeur K, Ball BA (2007). Characterization of NADPH oxidase 5 in equine testis and spermatozoa. Reproduction.

[28] Wong JL, Wessel GM (2005). Reactive oxygen species and Udx1 during early sea urchin development. Dev Biol.

[29] Guzik TJ, Chen W, Gongora MC, Guzik B, Lob HE, Mangalat D, Hoch N, Dikalov S, Rudzinski P, Kapelak B, Sadowski J, Harrison DG (2008). Calcium-dependent NOX5 nicotinamide adenine dinucleotide phosphate oxidase contributes to vascular oxidative stress in human coronary artery disease. J. Am. Coll. Cardiol..

[30] Zhu C, Yu ZB, Chen XH, Ji CB, Qian LM, Han SP (2011). DNA hypermethylation of the NOX5 gene in fetal ventricular septal defect. Exp. Ther. Med..

[31] Yuan X, Huang J, Wen L, Novakovic B, Kilby MD, Tong C, Qi H, Saffery R, Baker PN (2023). Genome-wide DNA methylation analysis of discordant monozygotic twins reveals consistent sites of differential methylation associated with congenital heart disease. Genomics.

[32] Enyedi B, Várnai P, Geiszt M (2010). Redox state of the endoplasmic reticulum is controlled by Ero1L-alpha and intraluminal calcium. Antioxid. Redox Signal..

[33] Schneider Z, Cervenak J, Baranyi M, Papp K, Prechl J, László G, Erdei A, Kacskovics I (2011). Transgenic expression of bovine neonatal Fc receptor in mice boosts immune response and improves hybridoma production efficiency without any sign of autoimmunity. Immunol. Lett..

