# Supplementary material for: A novel monoclonal antibody reveals the enrichment of NADPH oxidase 5 in human splenic endothelial cells
Source: Sci Rep. 2023 Oct 11;13:17174. doi: 10.1038/s41598-023-44018-5 (PMC10567692; doi:10.1038/s41598-023-44018-5)
Supplement: Supplementary file 1 — Supplementary Figures. [file 41598_2023_44018_MOESM1_ESM.pdf]

Supplementary figures

A NOVEL MONOCLONAL ANTIBODY REVEALS THE ENRICHMENT OF NADPH OXIDASE 5 IN HUMAN SPLENIC ENDOTHELIAL CELLS

Zsolt Szeles, Gábor L. Petheő, Bence Szikora, Imre Kacskovics, Miklós Geiszt

Supplementary figure 1.: Original Western blot and replicates for Figure 2.

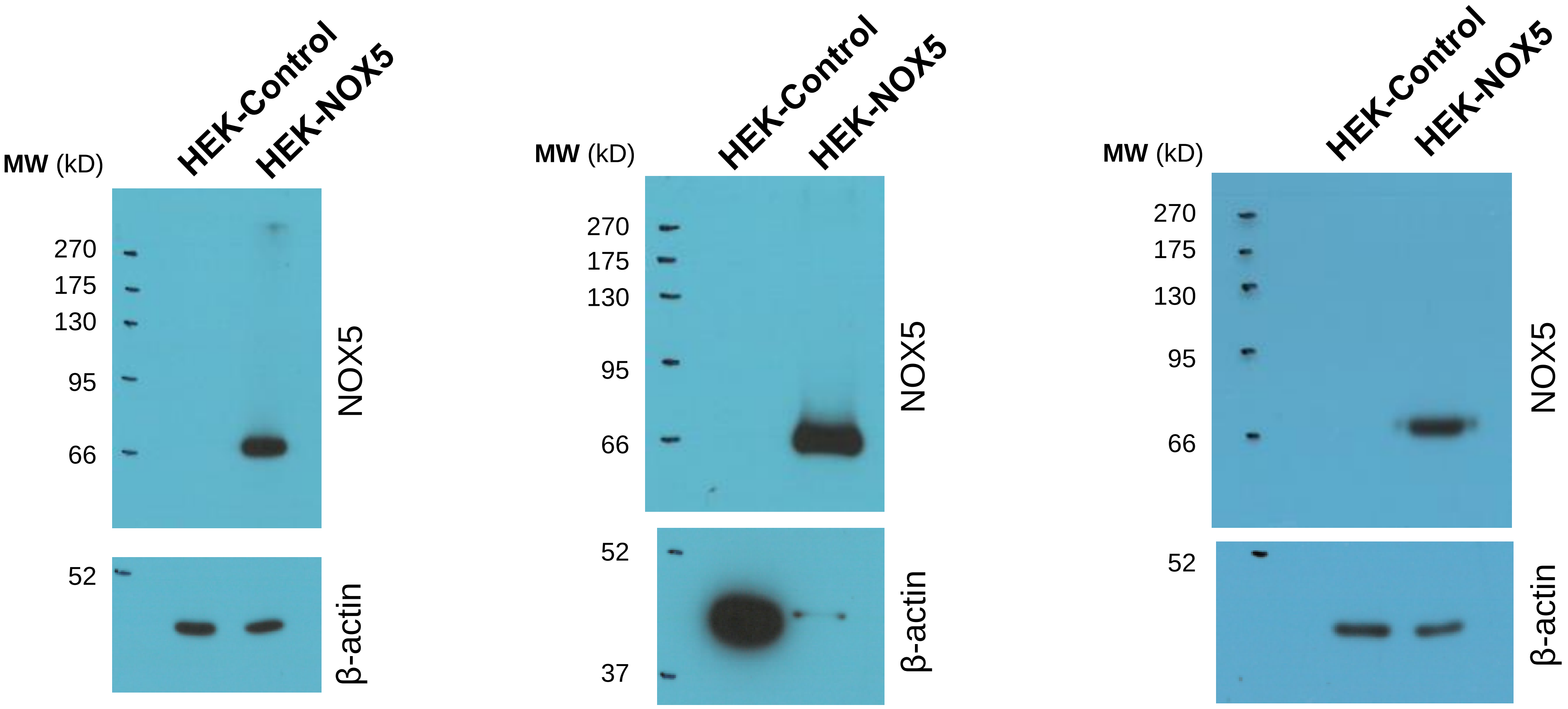

Detection of overexpressed NOX5 protein in HEK293T cell lysate by Western blot.

Supplementary figure 2.: Original Western blot and replicate for Figure 4.

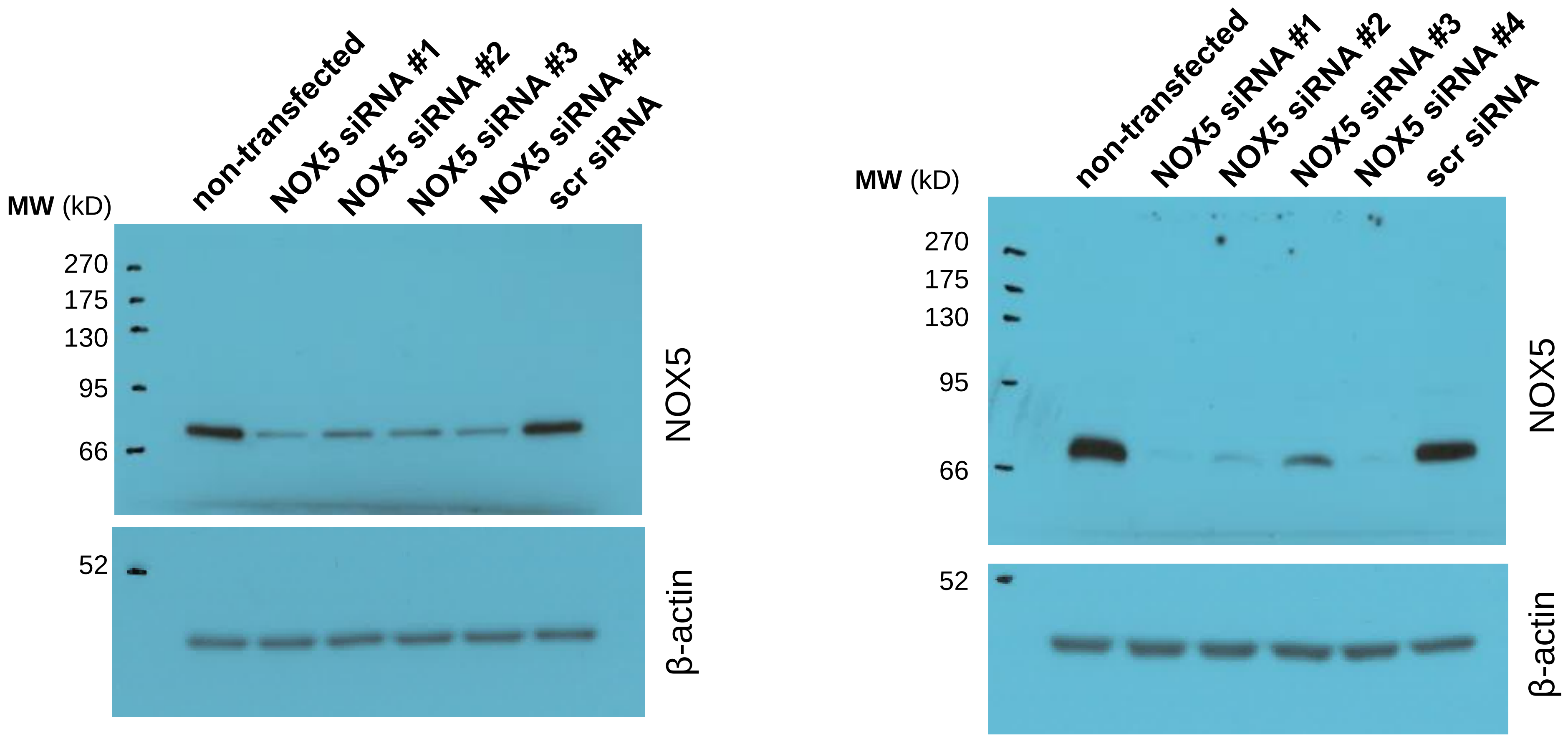

Validation of the novel NOX5 antibody on UACC-257 cells, using different UACC-257 cell lysates. The NOX5 protein expression was successfully attenuated by four different NOX5 siRNAs (NOX5 siRNA #1-#4), while the amount of NOX5 did not decrease in the scrambled (scr) siRNA transfected cells.

Supplementary figure 3.: Detection of NOX5 protein in UACC-257 cells using two different sets of molecular weight markers.

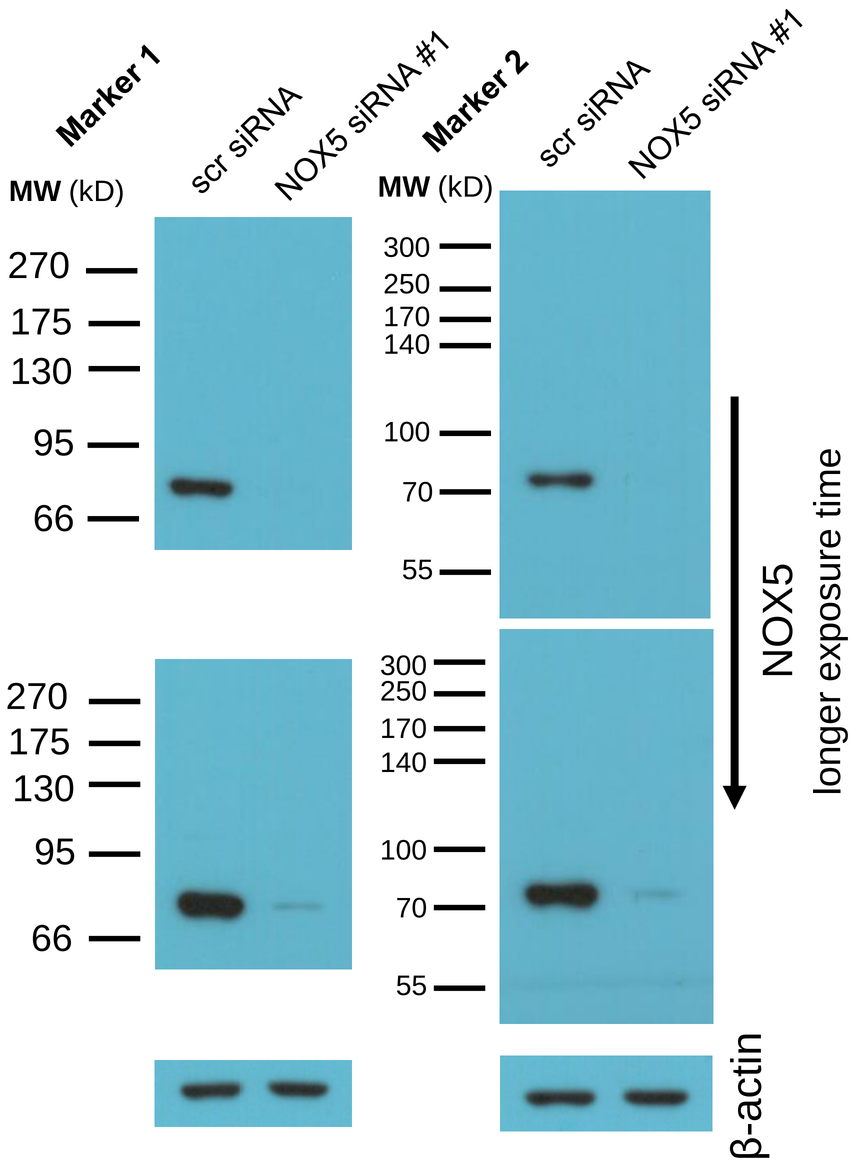

Two different protein markers were used to determine the molecular weight of NOX5 in UACC-257 cell lysates. As indicated by Marker 2, the molecular weight of NOX5 is around 75 kDa. The following Markers were applied: Marker 1: BioLegend Cat # 773302; Marker 2: Lonza: Catalog #: 00193837.

Supplementary figure 4.: Detection of endogenous NOX5 protein by Western blot in UACC-257 cell lysates with decreasing cell number.

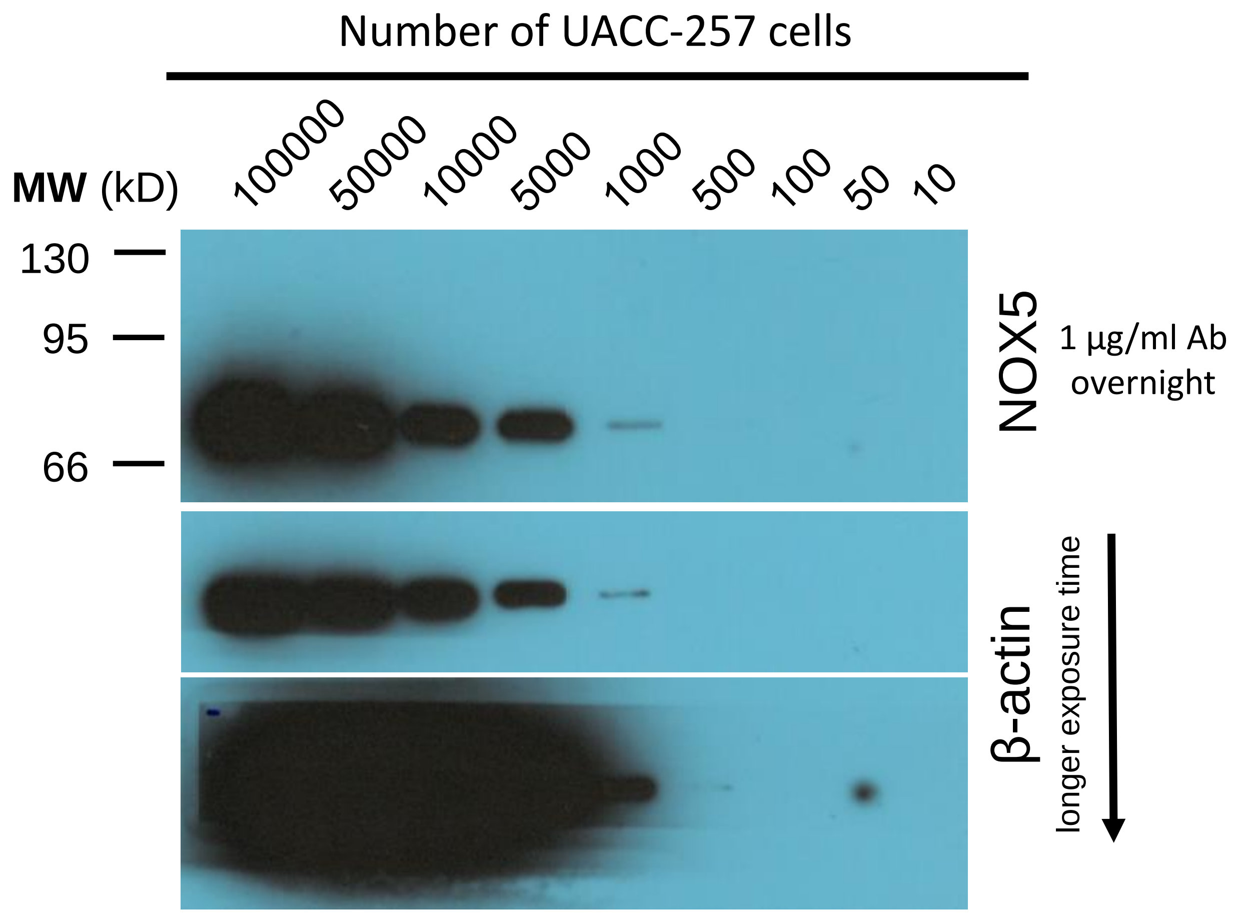

Detection of endogenous NOX5 protein in a given number of UACC-257 cells. The novel monoclonal NOX5 antibody was applied at a concentration of 1 µg/ml, overnight. The antibody detects the protein in as few as 1000 cells, although NOX5 is not uniformly expressed by the cells.

Supplementary figure 5.: Original Western blot and replicates for Figure 5.

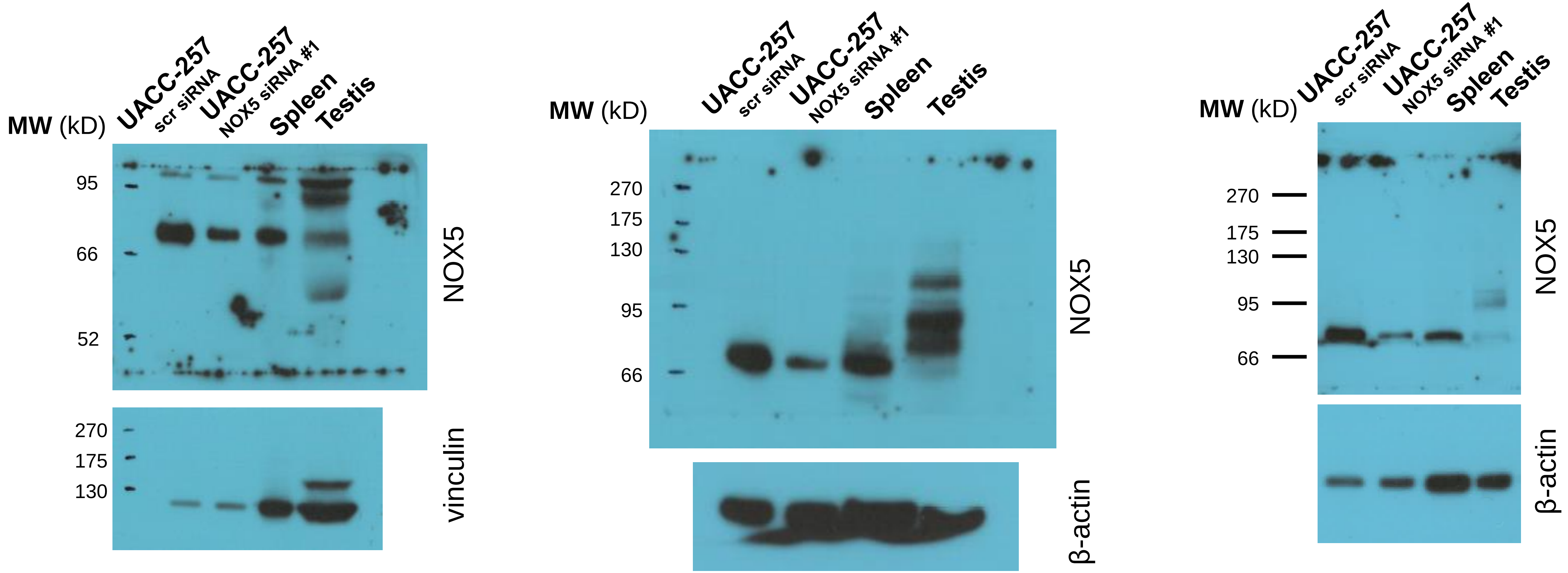

Detection of the NOX5 protein in human spleen and testis tissue lysates. To verify the specificity of the signal, scrambled (scr) and NOX5 siRNA transfected UACC-257 cell lysates were tested alongside with the tissues.

Supplementary figure 6.: Immunohistochemical detection of NOX5 protein in tissue section of human testis at 630x magnification.

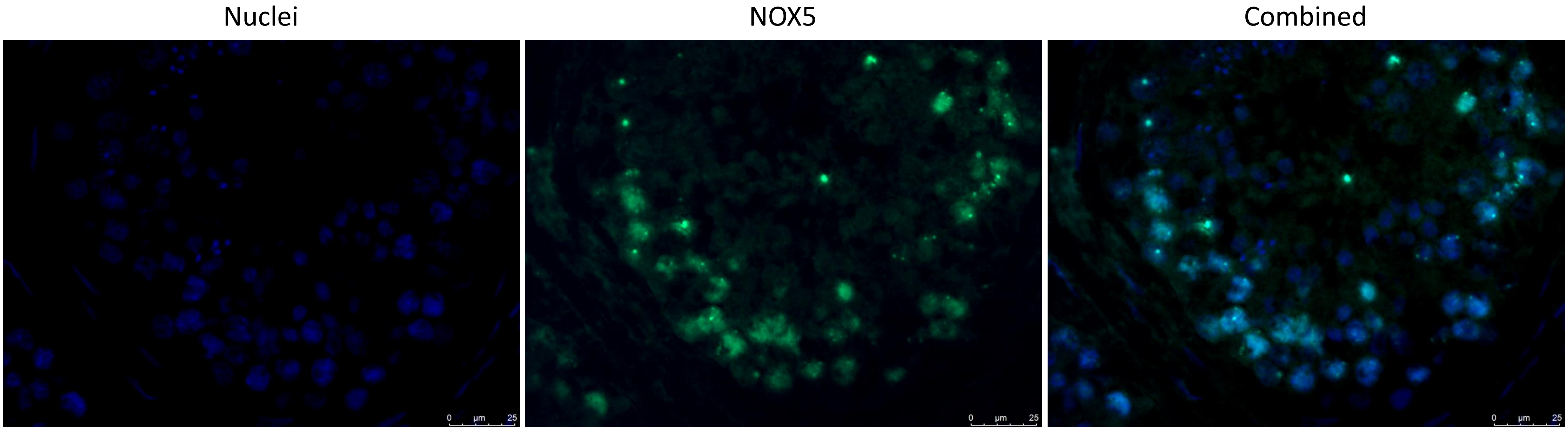

The green signals are associated with spermatogonic cells in the seminiferous tubules.

Supplementary figure 7.: Original Western blot and replicates for Figure 7.

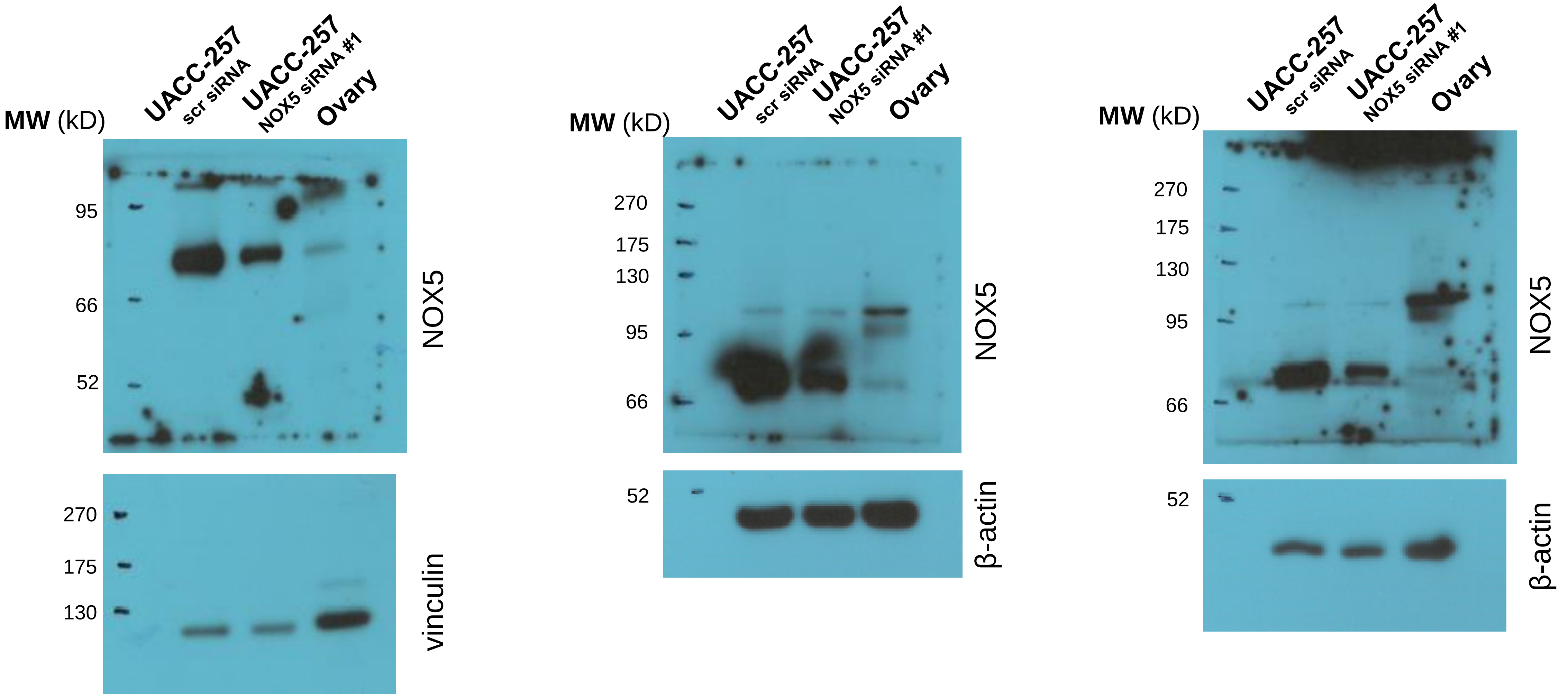

Detection of NOX5 protein in human ovary lysate. To verify the NOX5 specific signal, scrambled (scr) and NOX5 siRNA transfected UACC-257 cell lysates were tested alongside with the tissues.

Supplementary figure 8.: Original Western blot and replicate for Figure 8.

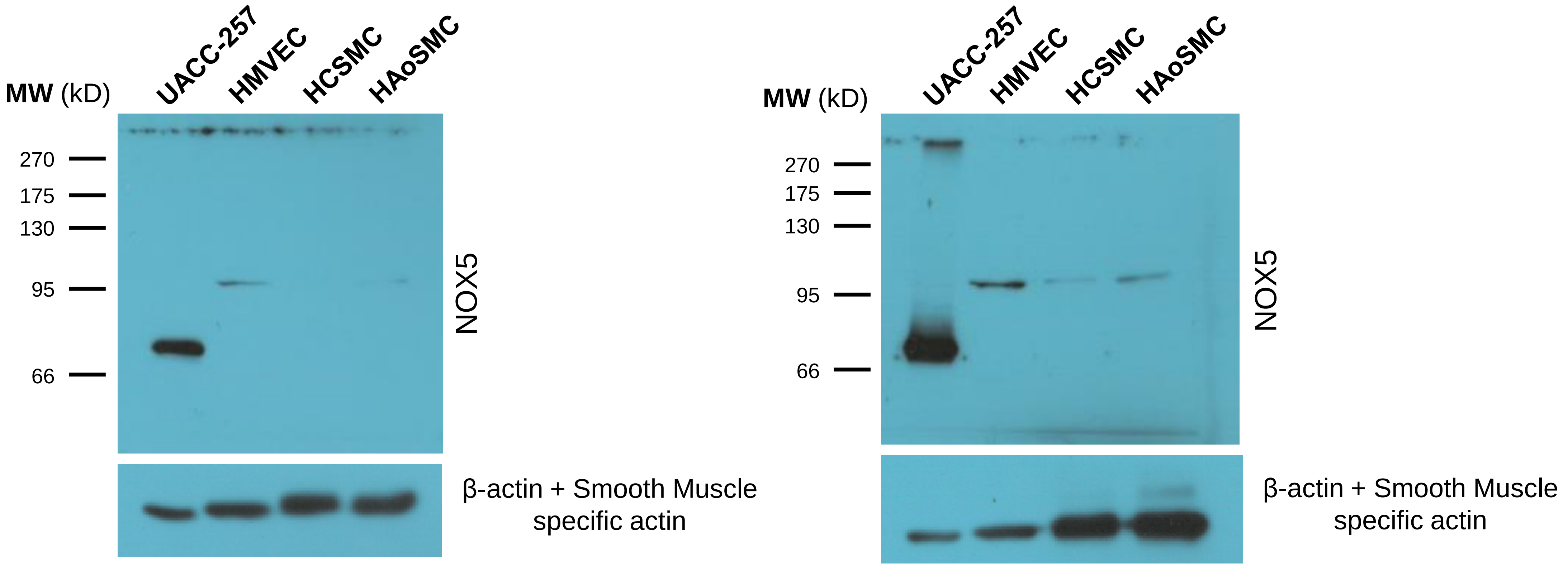

Detection of NOX5 protein in human primary vascular cells. To verify the identity of smooth muscle cells, an antibody specific for smooth muscle actin was applied. The different labels refer to the following samples: HMVEC: Human cardiac microvascular endothelial cells; HCSMC: Human coronary smooth muscle cells; HAoSMC: Human aortic smooth muscle cells.

Supplementary figure 9.: Control immunostainings for the detection of the NOX5 in the spleen

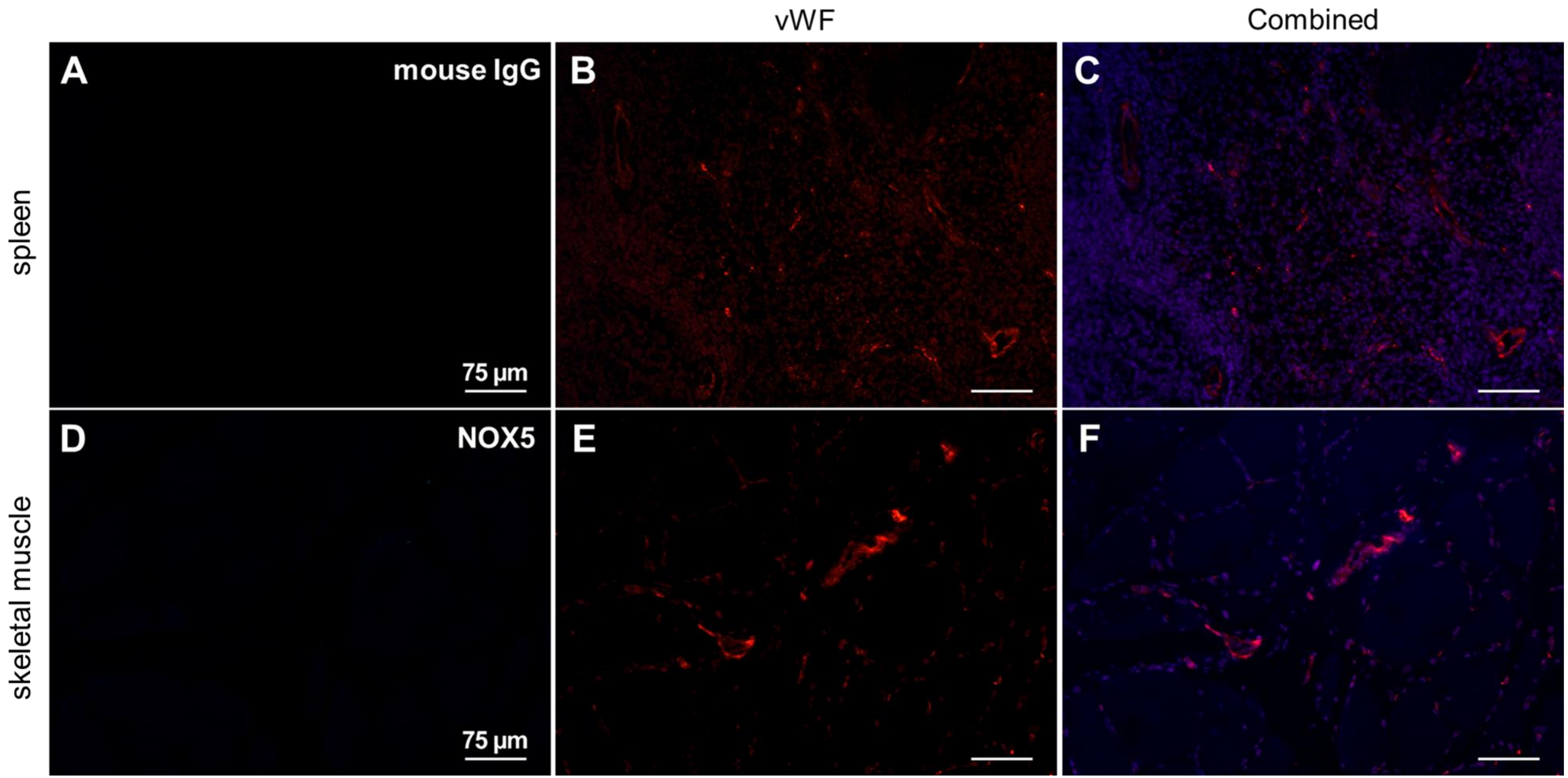

As antibody control, mouse IgG was used to stain human spleen tissue. **A** The tissue was incubated with mouse IgG. **B** vWF antibody was used to visualize the vessel structure **C** The combined picture from image A and B. The mouse IgG does not show any specific localization.
As a tissue control, human skeletal muscle tissue was used. The section was treated with the novel NOX5 (**D**) and the vWF (**E**) antibodies. Image **F** shows the merged signals from image D and E. The NOX5 antibody does not show an overlapping distribution with endothelial cells in the skeletal muscle.
